# Comparative microbial metagenomic analysis of drinking water plants and wastewater treatment plants in Istanbul

**DOI:** 10.3389/fmicb.2025.1488268

**Published:** 2025-01-20

**Authors:** Mehmet Demirci, Cankut Çubuk, Ferhat Dasdemir, Abdulkerim Suat Saribas, Esra Billur Balcıoglu, Dogukan Ozbey, Dogu Yorulmaz, Tugba Olmez Hanci, Safak Basa, Bekir Sami Kocazeybek

**Affiliations:** ^1^Department of Medical Microbiology, Faculty of Medicine, Kirklareli University, Kırklareli, Türkiye; ^2^Centre for Experimental Medicine and Rheumatology, William Harvey Research Institute, Queen Mary University of London and Barts NIHR BRC & NHS Trust, Charterhouse Square, London, United Kingdom; ^3^Department of Medical Microbiology, Faculty of Medicine, Istanbul University-Cerrahpaşa, Istanbul, Türkiye; ^4^Division of Marine Sciences and Management, Department of Chemical Oceanography, Istanbul University Institute of Marine Sciences and Management, Istanbul, Türkiye; ^5^Faculty of Medicine, Medical Faculty Student, Istanbul University-Cerrahpaşa, Istanbul, Türkiye; ^6^Istanbul Water and Sewerage Administration, Istanbul, Türkiye

**Keywords:** microbial metagenomic analysis, wastewater, drinking water, microbiome, antimicrobial resistance (AMR) gene

## Abstract

**Introduction:**

Wastewater treatment plants (WWTPs) and drinking-water treatment plants (DWTPs) are critical for public health due to the potential risks posed by microorganisms that may persist after treatment. The aim of this study was to detect the microbiome profiles of waters from both DWTPs and WWTPs under the Istanbul Water and Sewerage Administration (ISKI), identify the antimicrobial resistance profiles in all these facilities, and observe the differences in the microbiome between the inlet and outlet of different WWTPs.

**Methods:**

A total of 52 samples were examined, comprising 18 samples from DWTPs and 34 samples from WWTPs. All water samples underwent pre-isolation filtration. DNA isolation was conducted using filter material, followed by sequencing on a NovaSeq 6000 instrument. Kraken2 tools and R scripts were used for statistical analysis and data visualization.

**Results:**

The microbial metagenomic analysis identified 71 phyla, 113 classes, 217 orders, 480 families, and 1,282 genera across all samples. There were unclassified microbes (53.14% vs. 58.75%), Eukaryota (3.64% vs. 3.5%), Archaea (0.08% vs. 0.03%), bacteria (42% vs. 36.25%), and viruses (0.02% vs. 0.04%) in the raw water and ozonation unit outlet of DWTPs. The inlet and outlet of WWTPs showed unclassified microbes (52.68% vs. 59.62%), Eukaryota (0.6% vs. 1.72%), Archaea (0.26% vs. 0.15%), bacteria (46.43% vs. 38.43%), and viruses (0.05% vs. 0.04%). No statistically significant results were found in the analysis of raw waters collected from DWTPs and samples taken from the ozonation unit outlet—from the phylum level to the genus level (*p* > 0.05). The inlet and outlet points of WWTPs showed no statistically significant results from the phylum to species levels (*p* > 0.05). The most detected genera were *Desulfobacter* (4.82%) in preliminary WWTPs, *Thauera* (1.93%) in biological WWTPs, *Pseudomonas* (1.44%) in advanced biological WWTPs, *Acidovorax* (1.85%) in biological package WWTPs, and *Pseudomonas* (11.55%) in plant-based WWTPs. No antimicrobial resistance gene markers were detected in water samples from raw water inlets and ozonation unit outlets from DWTPs, membrane wastewater recovery plants, or ultraviolet (UV) recycling facilities. The *ANT*(3″), *Erm*, and *Sul* resistance gene markers were detected in all raw WWTPs samples.

**Discussion:**

There were no significant microbial risk differentiation between biological WWTPs and advanced biological WWTPs. The data could serve as preliminary information for future research. More extensive studies are needed, with multiple sample tracking in these facilities and their feeding basins.

## Introduction

Wastewater treatment plants (WWTPs) are critical points for public health due to the discharge of large volumes of treated wastewater into the environment after processing. However, they can release various infectious disease agents and resilient bacteria containing antimicrobial resistance (AMR) genes (ARGs) and associated mobile genetic elements after ineffective treatment procedures. Drinking water treatment plants (DWTPs) are similarly regarded as critical points for public health and have the potential to harbor microorganisms that may not be detected through routine assessments using conventional methods. Thus, incompletely treated water poses a risk of transmitting microorganisms to humans, much like WWTPs (Ng et al., 2019; Chu et al., 2018; Dias et al., 2020; Zieliński et al., 2022).

In Turkey and globally, there are no regulatory standards for tracking resistant microorganisms, antimicrobial concentrations, or ARGs in wastewater, and active surveillance in this regard is conducted at only a very limited number of sites (Mutuku et al., 2022). Poorly treated water from a treatment plant with substandard regulations can still transmit microorganisms, resistance genes, and mobile genetic elements. Following encounters with these microorganisms, other microorganisms that have acquired these resistance genes can spread among humans, which makes it necessary to monitor these waters (Chu et al., 2018). The World Health Organization (WHO) has set limits for drinking water and recommends monitoring DWTPs using conventional methods. The routine use of nucleic acid amplification-based techniques in monitoring can pose microbial risks due to the inability to detect viability, the potential for false positive or negative results, and their focus on a single target (requiring primers) (Novak Babič et al., 2020).

The “One Health” approach emerged from Rudolph Virchow’s idea in the 19th century that “there is no boundary between human and animal medicine, and there should not be.” Concerning infectious diseases, this approach emphasizes that not only humans but also other host organisms and all ecosystems that they inhabit could contribute to the spread of these pathogens, which highlights the importance of monitoring (Lira et al., 2020; Serpen, 2020; O'Brien and Xagoraraki, 2019). In WWTPs, waters are considered focal points for “One Health” monitoring due to the various microorganisms that they contain and their direct use for consumption in DWTPs (Ng et al., 2019; Sidhu et al., 2017).

Microorganisms are present throughout the planet, and describing all microorganisms and their functions was quite challenging until the advent of advanced next-generation sequencing (NGS) techniques in recent years. Microbial metagenomic analysis is one such technique that has facilitated detection of all microorganisms and their nucleic acids in any sample independently from classical culture methods (Brumfield et al., 2020). Alongside the heterogeneous mixture of microorganisms in waters entering WWTPs, dangerous metals and chemicals can also be present. Biological WWTPs use mixed groups of microorganisms in activated sludge to purify these waters and reduce public health risks (Sidhu et al., 2017). Microbial pollution may increase over time due to microorganisms entering these facilities and adapting to the changing ecosystem, as well as the various filtration techniques used in WWTPs. Thus, it is important to monitor these facilities and waters using new metagenomic techniques (De Celis et al., 2020).

Infectious diseases are considered one of the most critical threats to global public health. The emergence of new pathogenic organisms and the reemergence of once-controlled infectious agents are inevitable due to climate change and population growth (Lindahl and Grace, 2015). The ability to rapidly monitor the spread of these infectious diseases is key to their prevention, intervention, and control, yet current surveillance systems fall short in meeting this need (Aborode et al., 2021). Wastewater-based epidemiology studies using new molecular methods have been seen as a complementary approach in recent years for existing surveillance systems for infectious disease and could serve as an early warning system for potential outbreaks (Sims and Kasprzyk-Hordern, 2020; Bruno et al., 2022).

Taylor et al. (2024) conducted metagenomic analyses between the point of origin and distribution points in drinking water. They found that prominent bacterial families included nitrifying bacteria (Nitrospiraceae), iron-oxidizing bacteria (Gallionellaceae, Acidiferrobacteraceae), and Fe(III) metal-reducing bacteria (Geobacteraceae). Among the bacterial families examined, fewer than 1% were potentially pathogenic species, indicating that the water quality was generally good. Begmatov et al. (2024) conducted a metagenomic analysis of raw wastewater, activated sludge, and treated wastewater samples from large-scale WWTPs in Moscow. They identified hundreds of ARGs in raw wastewater that confer resistance to commonly used classes of antibiotics. They reported that resistance genes constitute approximately 0.05% of the metagenome and this rate decreased 3-to 4-fold after treatment.

Brumfield et al. (2020) used metagenomic analysis methods to determine the microbiological content of drinking water, through which a more accurate and detailed analysis was performed compared to traditional culture methods. Although bacteria such as Actinobacteria and Proteobacteria were detected, fecal indicator bacteria such as *Escherichia coli* or enterococci were not detected in drinking water. Sekizuka et al. (2022) conducted a metagenomic analysis of ARGs and heavy metal resistance genes in effluents from municipal WWTPs in Tokyo and reported that sulfonamide resistance genes were frequently detected.

The aim of the present study was to detect the microbiome profile on waters from both DWTPs and WWTPs. Plants under the Istanbul Water and Sewerage Administration (ISKI) were examined. We also aimed to determine the antimicrobial resistance profile in all these facilities and to observe the differences in the inlet and outlet microbiomes of different plants.

## Methods

This study was a prospective cross-sectional study. According to ISKI, the population served in Istanbul is estimated to be 16 million with around 7 million subscribers, and 3.2 million cubic meters of water are provided to the city daily. There are 24 DWTPs in Istanbul, with drinking water lines extending 23,000 km. The city has 90 WWTPs with lines totaling 18,500 km. The DWTPs and WWTPs included in the research represent approximately 90% of those in Istanbul.

### Sample collection

We collected raw water samples entering DWTPs, water samples subjected to oxidation after ozonation, raw water samples taken from the inlet and outlet points of WWTPs, treated wastewater, and samples of water recycled through ultraviolet (UV) disinfection after treatment in WWTPs. The study included 52 samples total: 18 from DWTPs and 34 from WWTPs. Information regarding the sampled DWTPs and WWTPs is presented in Supplementary Tables 1, 2.

**Figure 1:**
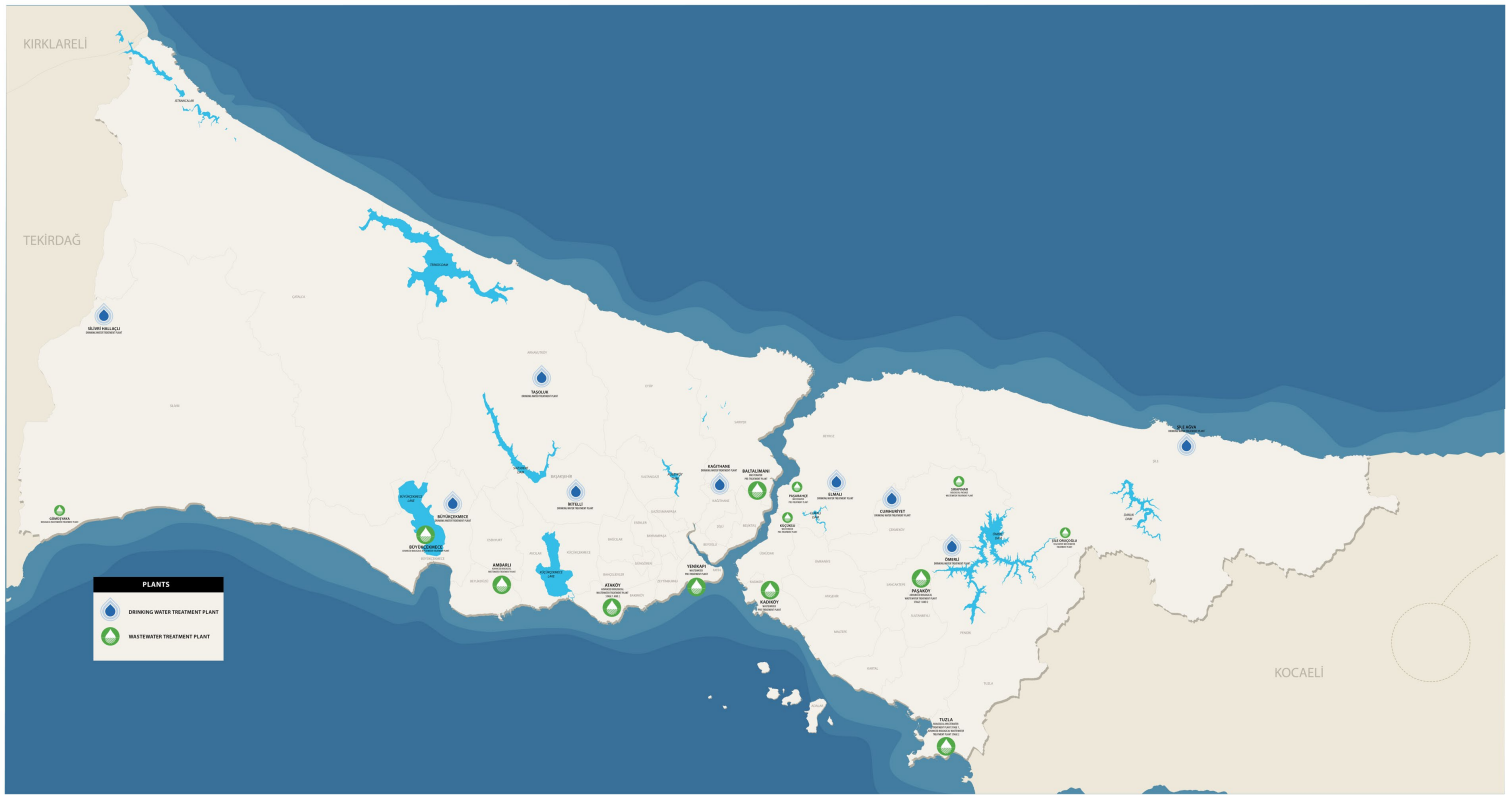
The location of the DWTPs and WWTPs included in our study.

The samples from DWTPs consisted of 13 raw water inlet samples and five samples from the ozone unit outlet (Table 1). A total of 34 wastewater inlet and outlet samples from 17 WWTPs were included. These comprised 10 samples from five different preliminary WWTPs, four samples from two distinct biological WWTPs, 14 samples from seven different advanced biological WWTPs, two samples from a biological package WWTP, two samples from a botanical (plant-based) WWTP, and two samples from a UV reclamation plant (Supplementary Table 2). The locations of the DWTPs and WWTPs included in the study are presented in Figure 1.

**Table 1:** Alpha diversity indexes for samples of DWTPs and WWTPs.

Alpha diversity index	DWTPs	WWTPs	*p*
Shannon	2.66 ± 0.93	4.7 ± 0.57	<0.05
Simpson	0.68 ± 0.18	0.94 ± 0.48	<0.05

Sampling was done using one-liter sterile polypropylene containers. After collection, samples were stored in foam containers with gel ice to maintain 4°C during transport to the laboratory.

### DNA isolation

In the laboratory, all water samples underwent pre-isolation filtration using Whatman filters and passage through a vacuum filtration setup. Samples were shaken before filtration. Next, samples were passed through a Whatman 47-mm glass microfiber filter (cat no: WHA1822-047) in the vacuum filtration device using a Rocker 300 oil-free vacuum pump at 670 mmHg (89.3 kPa). All of the Whatman filter paper material was treated with lysis buffer for isolation of DNA. Nucleogene Genomic DNA extraction kit were used for DNA isolation in these samples. (Cat no: NG041; Nucleogene Ltd., Kartal, Istanbul). All obtained DNA samples were checked for their concentrations using Qubit 4 Quantitation Starter Kit on a Qubit 4 Fluorometer (Thermo Scientific, CA, USA) according to manufacturer’s instructions.

### Next-generation sequencing

The DNA was subjected to library preparation. Following isolation, DNA fragments in each DNA sample were enzymatically reduced to lengths readable on sequencing platforms. This step was conducted using an Illumina DNA Prep kit (Illumina, USA, #20060059) following manufacturer protocols. During library preparation, IDT adapters for Illumina® DNA/RNA UD Indexes Set C (Illumina, USA, #20042666) were used for indexing the samples and adding Illumina index and adapter sequences. After polymerase chain reaction (PCR), the prepared library was measured again using a Qubit 4 Fluorometer and normalized before sequencing. Pair-end (2×150 bp) sequencing was carried out according to the guidelines of the NovaSeq 6000 next-generation sequencing platform (Illumina, USA) using NovaSeq 6000 S4 Reagent kit (Illumina, USA, #20028312).

### Bioinformatic analysis

FastQC v0.11.9 (Andrews, 2010) was used to assess the quality of raw sequence data. After quality control, raw reads were trimmed to remove low-quality base calls and Illumina adapters using the Trimmomatic tool (v. 0.39) with default parameters. Initial sequencing generated 12–17 million reads per sample. After these steps, the final usable sequencing depth ranged from 11 to 16 million paired-end reads per sample, averaging 14 million reads (Supplementary material). The datasets can be downloaded from https://www.ebi.ac.uk/biostudies/arrayexpress/studies/E-MTAB-14353.

For taxonomic profiling, reads were aligned to target organisms using the Kraken2 tool (v2.0.8) with the PlusPF database (Standard Plus Protozoa & Fungi; 12/9/2022). We used standard Kranken2 settings with modified parameters: confidence: 0.0, minimum base quality: 0, minimum hit groups: 2, and “use names.” Microorganism groups in each sample were determined after alignment, and KronaTools v2.7.1 was used to generate interactive plots exploring metagenome composition. The library R::vegan v2.7–0 was used to calculate diversity indices, and the R programming language (v4.2.0) was used to create data visualizations and perform statistical comparisons of groups using the t-test.

### ARGs identification

The Explify UPIP Data Analysis tool v7.3.6 (Illumina, USA) was used to detect pathogenic bacteria and their associated ARG markers. This tool enables simultaneous detection of more than 3,700 antimicrobial resistance markers, including rapidly or slowly growing aerobic and anaerobic uropathogens and sexually transmitted microorganisms. Furthermore, it enables comparison of the data with a database to extract profiles specific to the samples.

## Results

The microbial metagenomic analysis identified 71 phyla, 113 classes, 217 orders, 480 families, and 1,282 genera across all samples. The most prominently observed phylum was Proteobacteria in WWTPs (median = 25.6%, mean = 27.4, standard deviation (SD) = 8.5), DWTPs (median = 28.4%, mean = 34.2, SD = 18.9), and overall (median = 26.2%, mean = 29.8, SD = 13.3). The Shannon index and the Simpson index were used to assess the variety of species in the community and consider both the number of different species (richness) and how evenly spread out the individuals are among those species (evenness). Generally, a higher Shannon index and a lower Simpson index indicate a more diverse community. Table 1 shows the results of the Shannon and Simpson indexes in samples of DWTPs and WWTPs.

Microbial diversity, assessed by Shannon and Simpson indices, varied across different types of WWTPs. No significant differences in microbial communities were found between raw water and ozonated water in DWTPs. Some differences in microbial communities were observed between different types of WWTPs, but overall, the communities were relatively similar. Proteobacteria was the most abundant phylum and *Pseudomonas* was a dominant genus in both DWTPs and WWTPs. Other dominant genera varied depending on the type of treatment plant. No ARGs were detected in DWTPs. Some WWTPs (biological, advanced biological, and plant-based) effectively reduced the presence of ARGs.

### Metagenomic analysis of DWTPs

**Figure 2:**
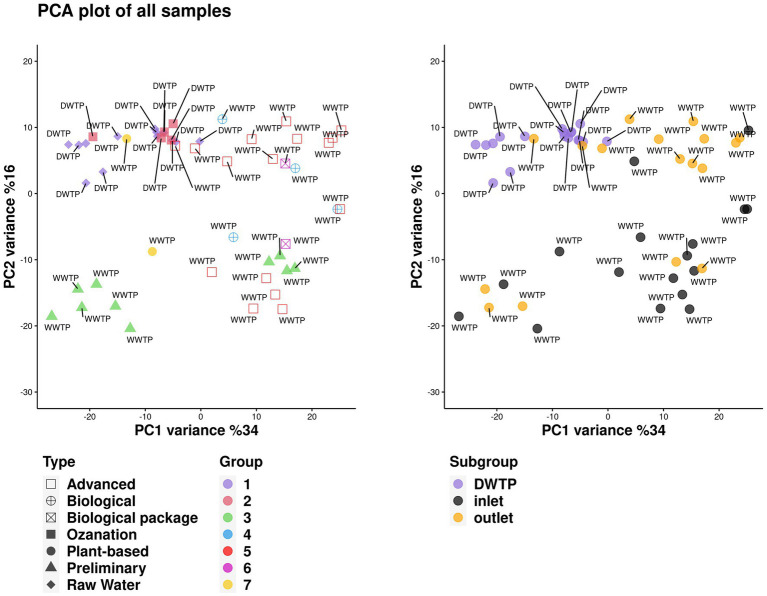
Distribution of all the samples with different categories on the PC1 and PC2 dimensions.

Figure 2 illustrates the results of principal component analysis (PCA). No statistically significant results were found in the microbial metagenomic comparative analysis of raw waters collected from DWTPs and samples taken from the ozonation unit outlet from the phylum level to the genus level (*p* > 0.05). Unclassified microbes (53.14% vs. 58.75%), Eukaryota (3.64% vs. 3.5%), Archaea (0.08% vs. 0.03%), bacteria (42% vs. 36.25%), and viruses (0.02% vs. 0.04%) were detected in the raw water and ozonation unit outlet in DWTPs, respectively.

**Figure 3:**
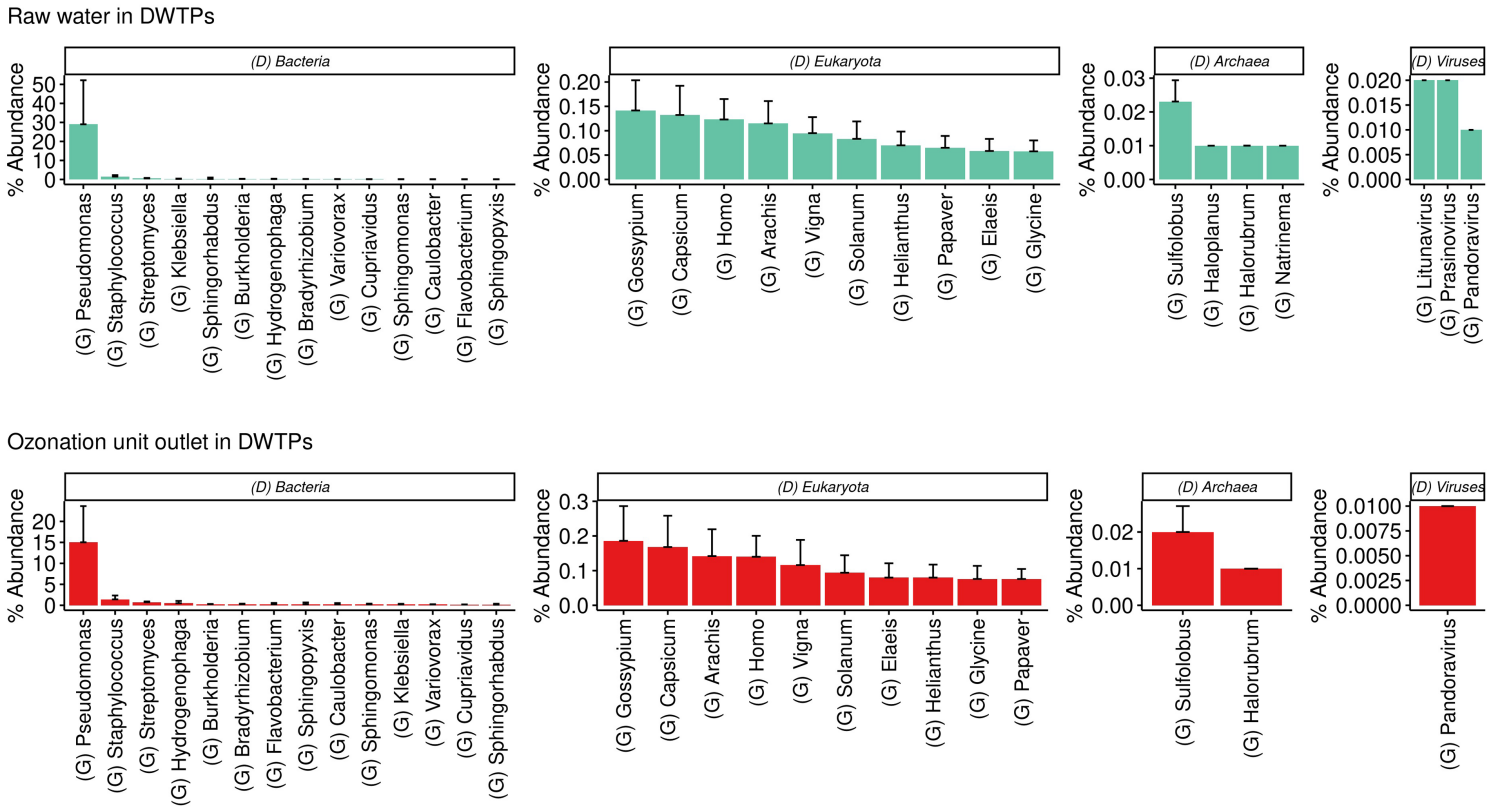
Barplots show the most abundant genera found in raw waters and samples from the ozonation unit outlet of DWTPs across several domains and viruses.

The dominant genera detected in the raw water were *Gossypium* for Eukaryota; *Sulfolobus* for Archaea; *Pseudomonas* for bacteria; and *Litunavirus* for viruses. The dominant genera detected at the ozonation unit outlet in the DWTPs were *Gossypium* for Eukaryota; *Sulfolobus* for Archaea; *Pseudomonas* for bacteria; and *Pandoravirus* for viruses. The sample krona plots obtained from these facilities are presented in Figure 3.

### Metagenomic analysis of WWTPs

Unclassified microbes (52.68% vs. 59.62%), Eukaryota (0.6% vs. 1.72%), Archaea (0.26% vs. 0.15%), bacteria (46.43% vs. 38.43%), and viruses (0.05% vs. 0.04%) were detected at the inlet and outlet of WWTPs, respectively. The inlet and outlet of preliminary (pre-treatment) WWTPs showed unclassified microbes (51.8% vs. 53.4%), Eukaryota (0.74% vs. 0.68%), Archaea (0.198% vs. 0.18%), bacteria (47.2% vs. 45.8%), and viruses (0.062% vs. 0.058%), respectively. In biological WWTPs, unclassified microbes (58% vs. 66%), Eukaryota (0.45% vs. 1.9%), Archaea (0.45% vs. 0.15%), bacteria (41% vs. 32%), and viruses (0.025% vs. 0.025%) were detected at the inlet and outlet, respectively. In advanced biological WWTPs, unclassified microbes (50.57% vs. 62.71%), Eukaryota (0.54% vs. 2.04%), Archaea (0.21% vs. 0.15%), bacteria (48.71% vs. 34.85%), and viruses (0.052% vs. 0.025%) were detected at the inlet and outlet, respectively. In biological package WWTPs, unclassified microbes (55% vs. 55%), Eukaryota (0.4% vs. 1%), Archaea (0.3% vs. 0.1%), bacteria (44% vs. 44%), and viruses (0.03% vs. 0.02%) were detected at the inlet and outlet, respectively. In plant-based WWTPs, unclassified microbes (59% vs. 61%), Eukaryota (0.9% vs. 5%), Archaea (0.5% vs. 0.08%), bacteria (40% vs. 34%), and viruses (0.03% vs. 0.08%) were detected at the inlet and outlet, respectively.

**Figure 4:**
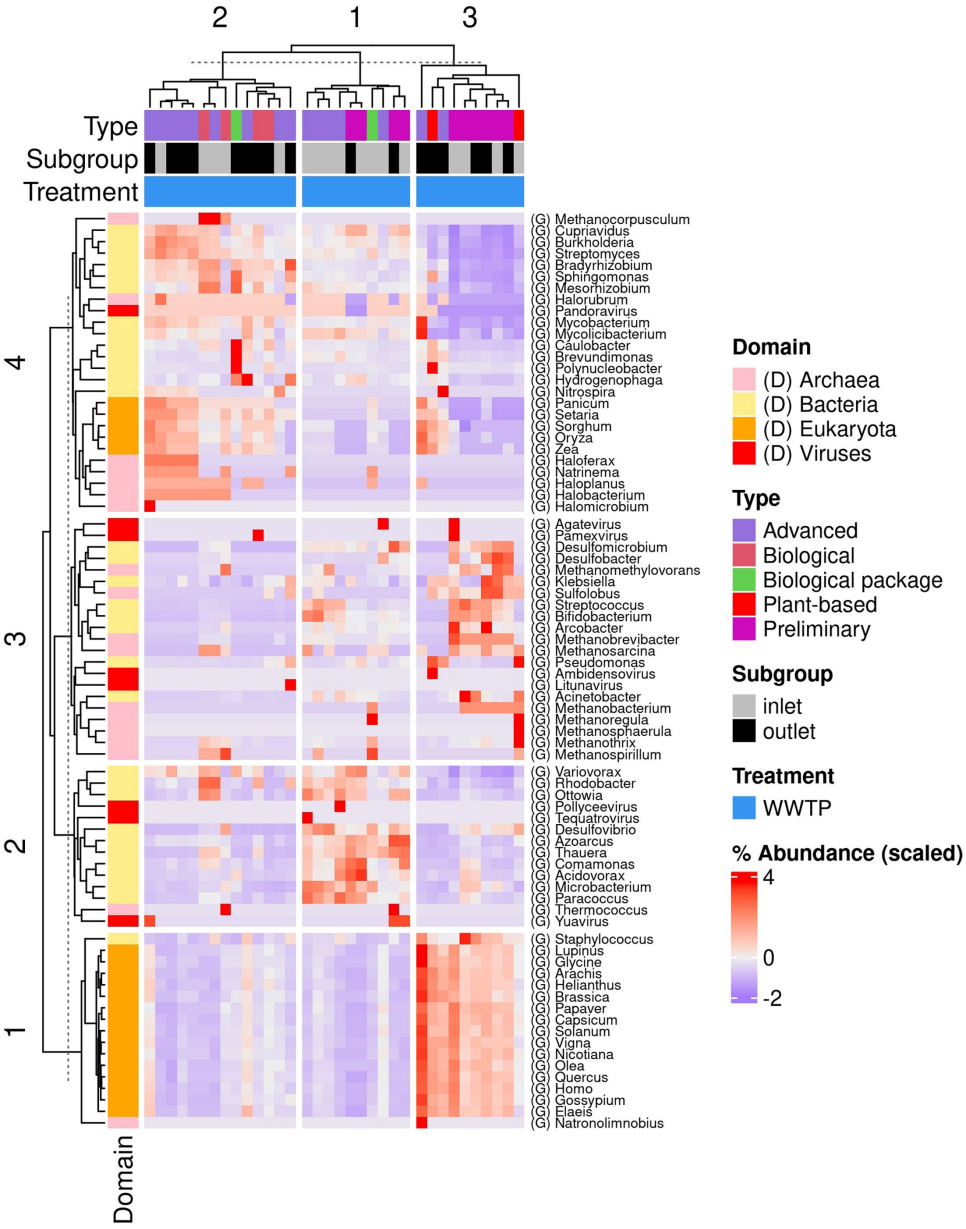
Heatmap shows the dominant genera detected in the WWTPs across different domains and viruses.

The dominant genera detected at the inlet of the WWTPs were *Leishmania*, *Saccharomyces*, and *Plasmodium* spp. for Eukaryota; *Methanobrevibacter*, *Halorubrum*, and *Methanotrix* for Archaea; *Desulfobacter*, *Acidovorax*, *Thauera*, and *Pseudomonas* for bacteria; and *Crassphage*, *Pamexvirus*, and *Yuavirus* for viruses. At the inlet of the WWTPs, the dominant genera detected were *Leishmania*, *Saccharomyces*, and *Pyricularia* spp. for Eukaryota; *Methanobrevibacter*, *Halorubrum*, *Halosimplex*, and *Methanotrix* for Archaea; *Desulfobacter*, *Acidovorax*, *Streptomyces*, *Hydrogenophaga*, *Thauera*, and *Pseudomonas* for bacteria; and *Crassphage*, *Pamexvirus*, *Yuavirus*, *Nipunavirus*, and *Kagunavirus* for viruses (Figure 4). The Supplementary material shows the krono plots for each sample (Supplementary File).

**Figure 5:**
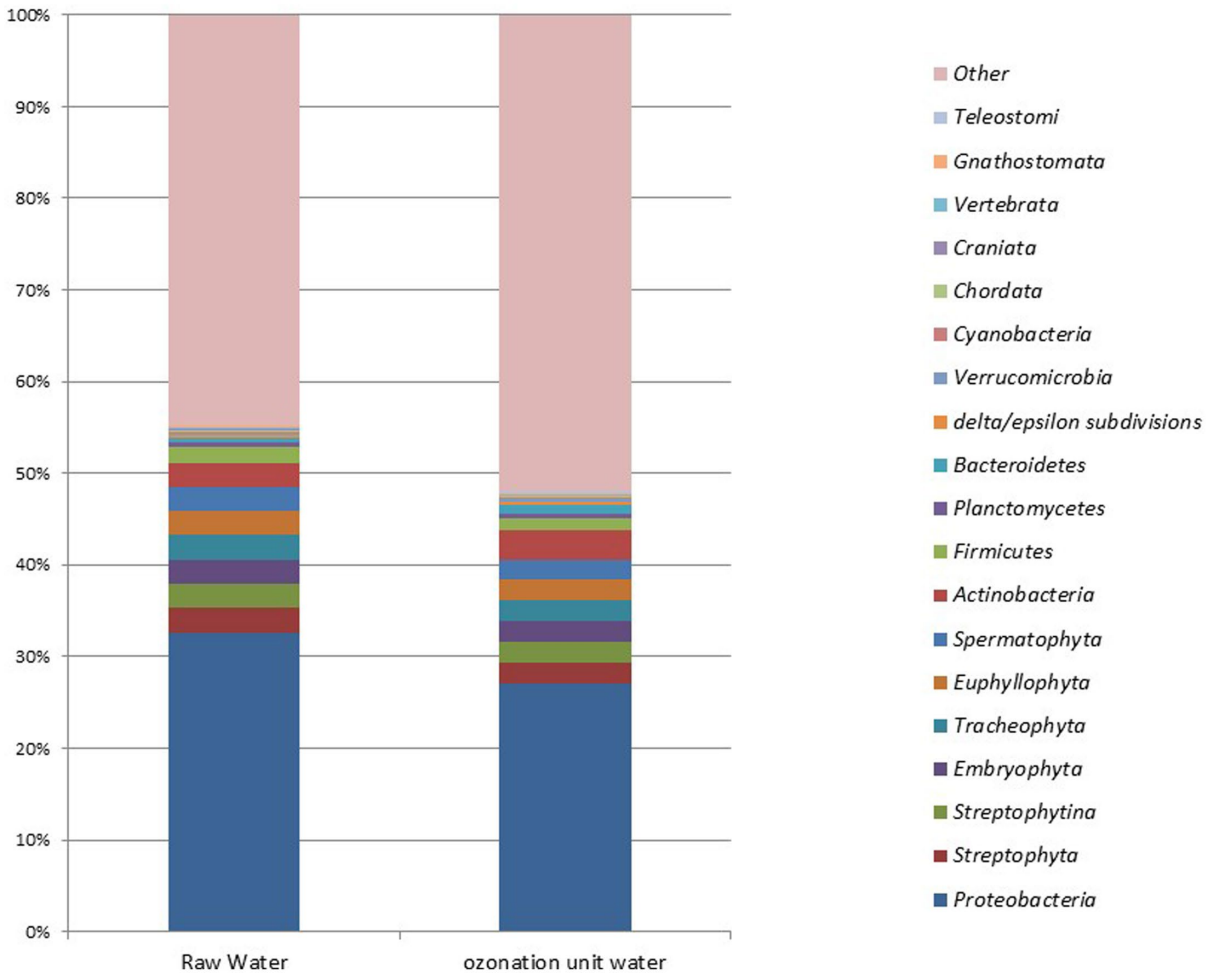
Microbial metagenomic comparison of the top 20 most detected phyla in raw waters from DWTPs and samples from the ozonation unit outlet.

**Figure 6:**
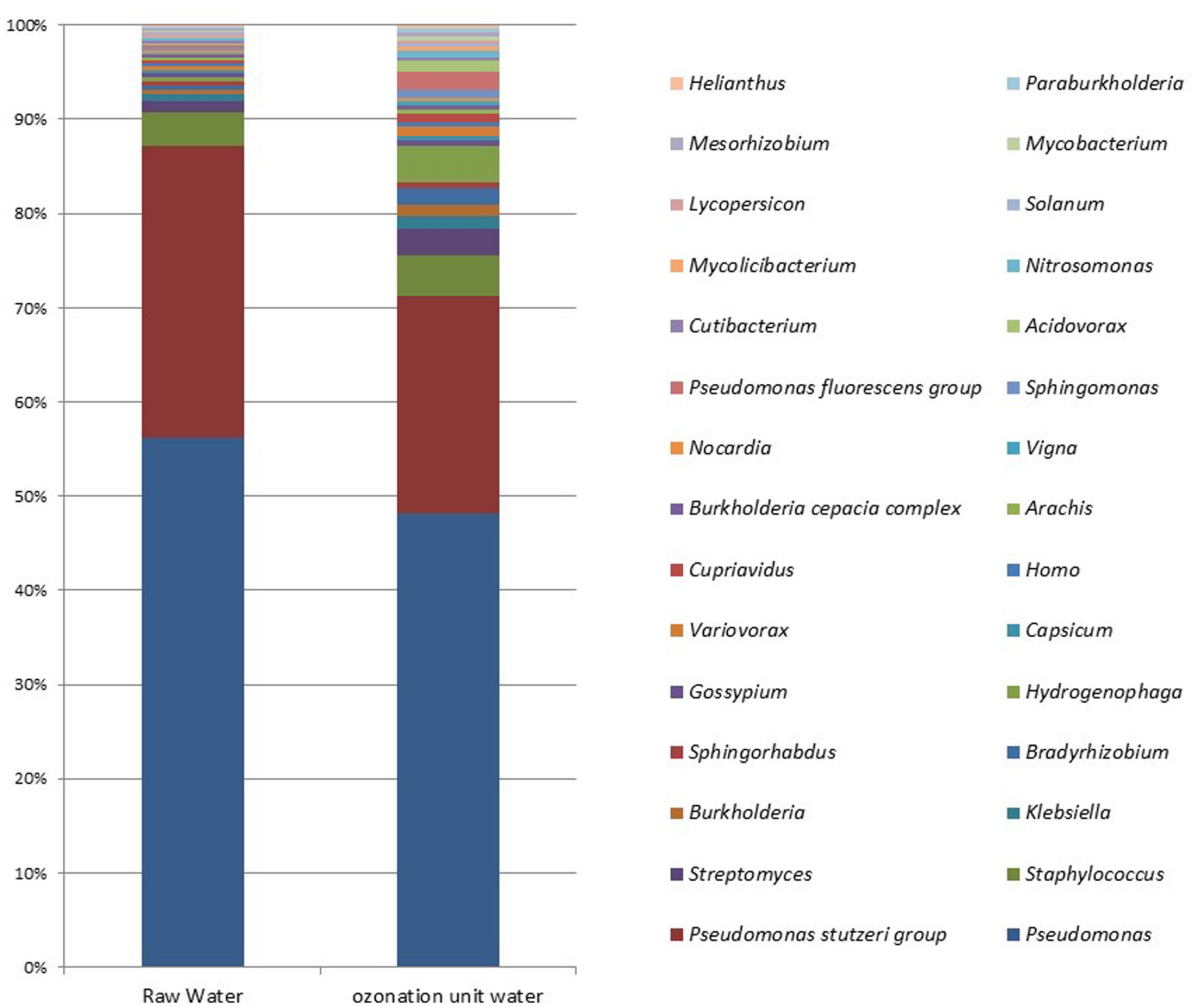
Microbial metagenomic comparison among the top 30 most detected genera in samples from raw waters of DWTPs and the ozonation unit outlet.

The average entry rates of dominant bacterial species in DWTPs were 42%, but the average decreased to around 36.25% at the ozonation outlet. At the species level, statistically significant differences were observed in *Novosphingobium* sp. P6W (*p* = 0.003), *Methylobacterium mesophilicum* (*p* = 0.02), *M. mesophilicum* SR1.6/6 (*p* = 0.02), and *Mesorhizobium* sp. 8 (*p* = 0.04). Statistically significant increases were detected in these species after ozonation compared to their amounts in raw waters (*p* < 0.05). Figure 5 illustrates the microbial metagenomic comparison between raw waters and samples taken from the ozonation unit at the phylum level, while Figure 6 shows the comparison at the genus level for the most highly detected phyla and genera. The most highly detected phylum was *Proteobacteria* (32.64 and 27.085%) in raw waters and samples taken from the ozonation unit outlet in DWTPs; the most common genus was *Pseudomonas* (24.33 and 11.87%) (Figures 5, 6).

The average entry rate of dominant bacterial species in DWTPs was 42%--this decreased to around 36.25% at the ozonation outlet. The rates were 24.33, 1.55, and 0.53% for the common genera *Pseudomonas*, *Staphylococcus*, and *Streptomyces* at the entrance of the water treatment facility, respectively. At the ozonation outlet, the rates were 11.87, 1.055, and 0.705% (Figure 6). The microbial metagenomic comparison of raw and treated wastewater samples from the inlet and outlet points of WWTPs showed no statistically significant results from phylum to species levels (*p* > 0.05). However, there were some differences at the order and family levels. The only statistically significant difference between the inlet and outlet samples was a decrease in the fungal order *Hypocreales* (*p* = 0.049) and an increase in the bacterial family *Christensenellaceae* (*p* = 0.049).

**Figure 7:**
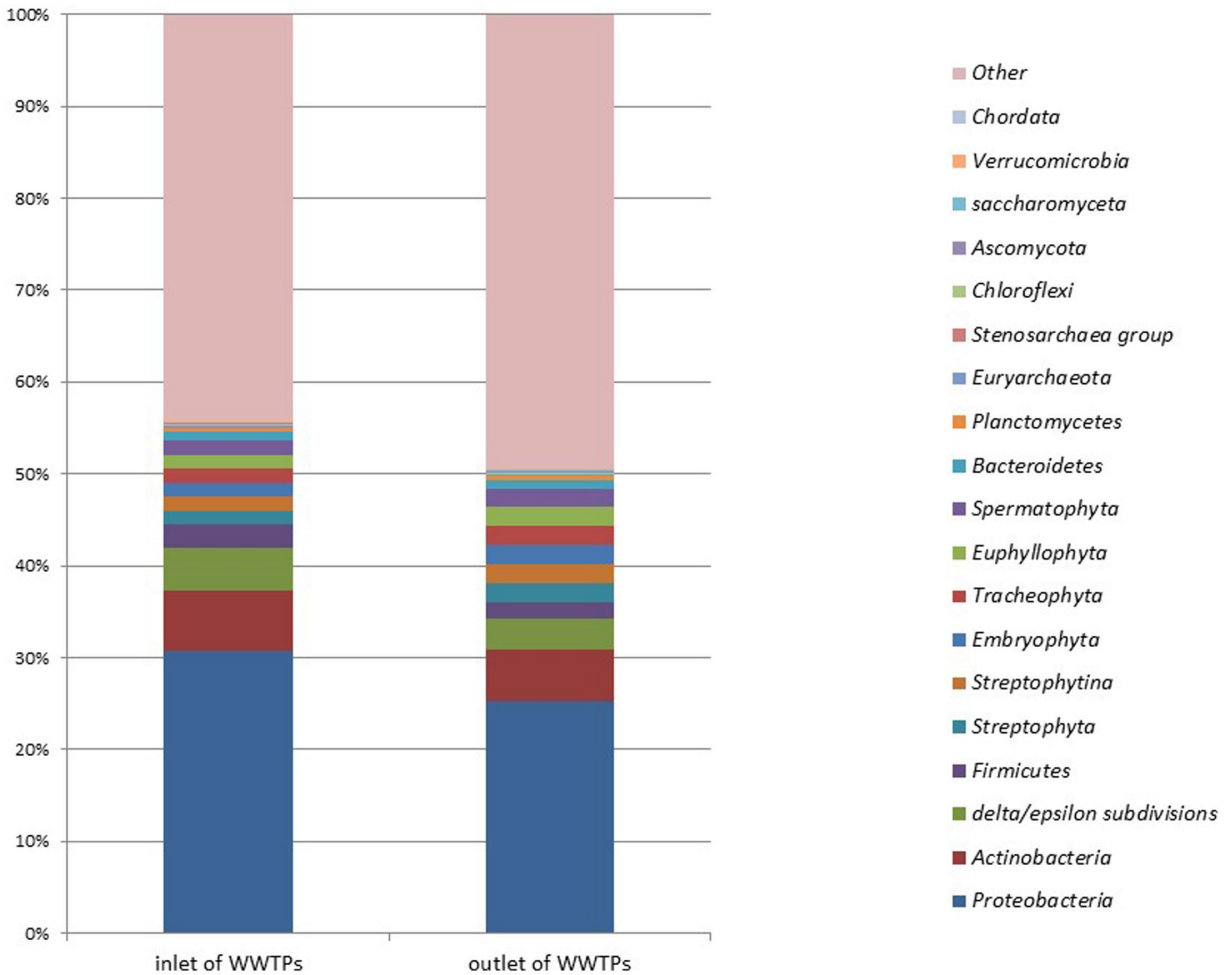
Microbial metagenomic comparison at the phylum level of samples taken from the inlet and outlet of WWTPs.

**Figure 8:**
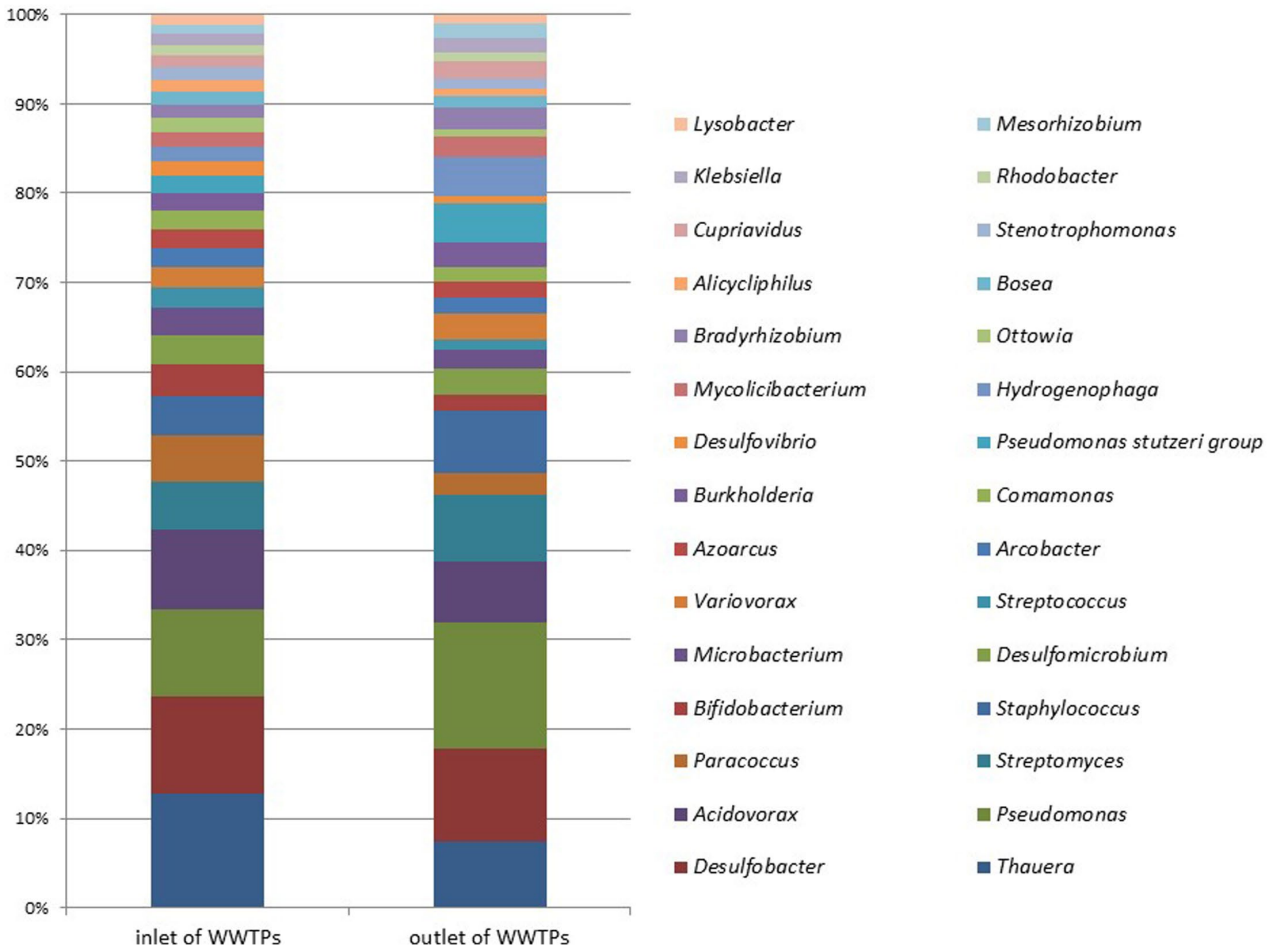
Microbial metagenomic comparison among the top 30 genera most detected in samples taken from the inlet and outlet of WWTPs.

Figure 7 illustrates the microbial metagenomic comparison at the phylum level, while Figure 8 demonstrates the comparison at the genus level for samples collected from the inlet and outlet of WWTPs. When comparing data obtained from preliminary WWTPs to biological WWTPs, the phylum *Proteobacteria* represented 31.25 and 23.93% of the respective isolates (*p* > 0.05). At the phylum level, significant differences were only detected in *Acidobacteria* (0.026 and 0.06%; *p* = 0.013).

At the genus level, significant differences were found between preliminary WWTPs and biological WWTPs in *Myxococcus* (0.021 and 0.04%; *p* = 0.03), *Rothia* (0.012 and 0.0001%; *p* = 0.005), *Micromonas* (0.005 and 0.02%; *p* = 0.005), and *Halorubrum* (0.001 and 0.01%; *p* = 0.005). A significant increase was observed in biological WWTPs compared to preliminary WWTPs in these phyla and genera except for *Rothia*, which showed a significant decrease. When comparing data obtained from preliminary WWTPs to advanced biological WWTPs, significant differences were observed in nine phyla (*Planctomycetes*, *Verrucomicrobia*, *Pezizomycotina*, *Leotiomyceta*, *Sordariomyceta*, *Acidobacteria*, *Euglenozoa*, *Chlorophyta*, and *Basidiomycota*) (*p* < 0.05). Significant differences were found between preliminary WWTPs and advanced biological WWTPs in 22 genera: (*Bradyrhizobium*, *Mycobacterium*, *Sphingomonas*, *Nocardioides*, *Rhodopseudomonas*, *Frankia*, *Phreatobacter*, *Nonomuraea*, *Methylorubrum*, *Microvirga*, *Hypericibacter*, *Methylocystis*, *Curtobacterium*, *Rothia*, *Pyricularia*, *Leishmania*, *Sphingosinicella*, *Cellulosimicrobium*, *Skermanella*, *Methylosinus*, *Kribbella*, and *Halorubrum*) (*p* < 0.05). A significant increase was observed in advanced biological WWTPs compared to preliminary WWTPs in these phyla and genera except for *Rothia*.

When comparing data from preliminary WWTPs to biological package WWTPs, significant differences were observed between the facilities in three phyla (*Pezizomycotina*, *Leotiomyceta*, and *Sordariomyceta*) (*p* < 0.05). In biological package WWTPs, there was a significant increase in these phyla versus preliminary WWTPs. At the genus level, significant differences were detected in 11 genera (*Rathayibacter*, *Setaria*, *Curtobacterium*, *Planctomyces*, *Microterricola*, *Rothia*, *Frondihabitans*, *Micromonas*, *Phycicoccus*, *Luteipulveratus*, and *Halorubrum*) (*p* < 0.05). These phyla and genera (except for *Rothia*) showed a significant increase in biological package WWTPs compared to preliminary WWTPs. However, a significant decrease was noted in *Rothia*.

Significant differences were only observed in phylum *Actinobacteria* (5.09 and 2.4%; *p* = 0.014) when comparing preliminary WWTPs to plant-based biological WWTPs. At the genus level, significant differences were detected in five genera (*Parolsenella*, *Gordonibacter*, *Rothia*, *Eggerthella*, and *Miniimonas*) (*p* < 0.05). No significant differences were observed at any level when comparing data from biological WWTPs to advanced biological WWTPs (*p* > 0.05). The most common phylum was *Proteobacteria*. The proportions of *Proteobacteria* were 23.93 and 25.91% in biological WWTPs and advanced biological WWTPs, respectively.

**Figure 9:**
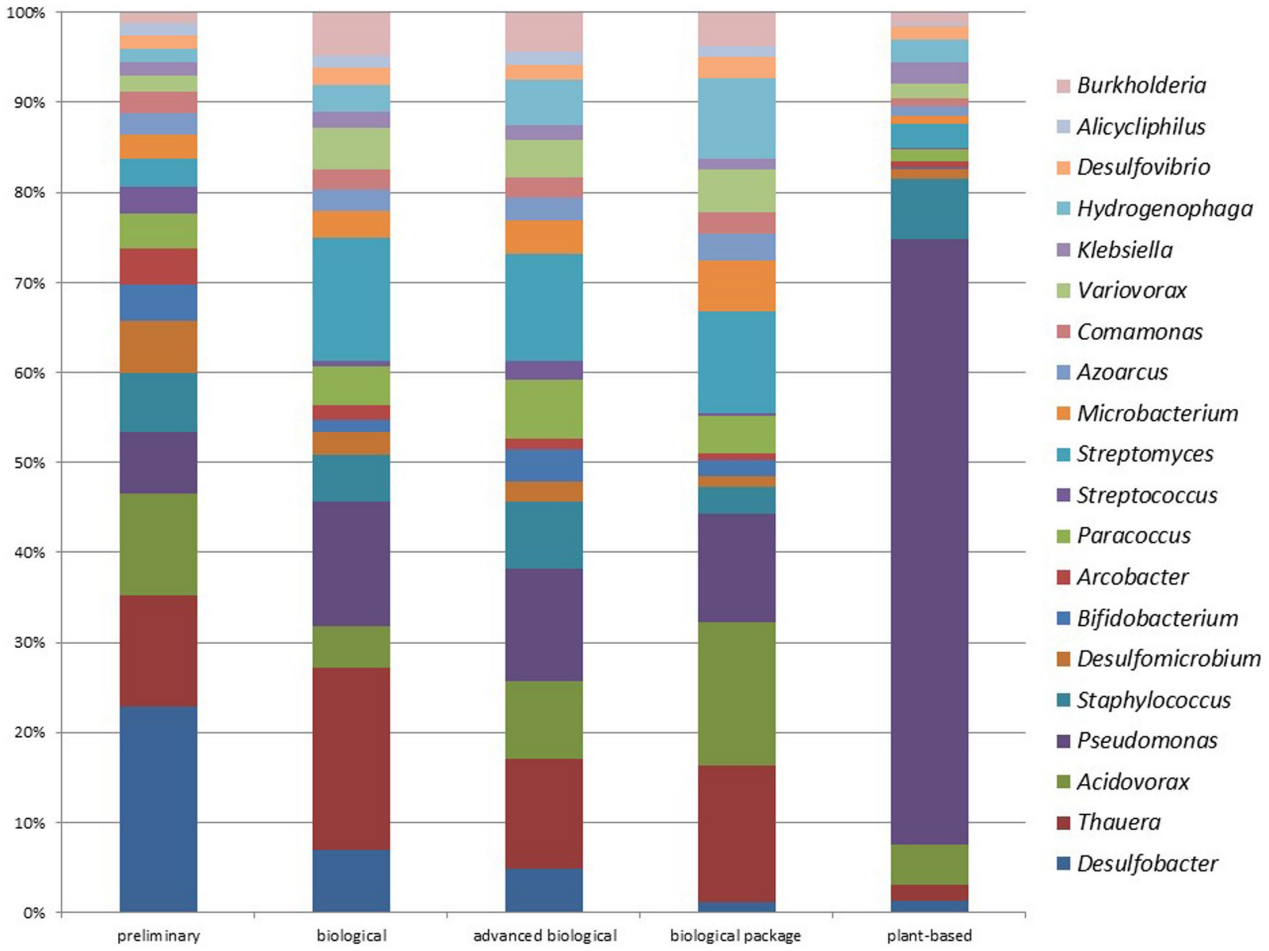
Comparison of the top 20 genera from different WWTPs (Preliminary: preliminary WWTPs; Biological: Biological WWPTs; advanced biological: advanced biological WWTPs; biological package: package WWTPs; plant-based: Vegetation-based WWTPs).

In biological WWTPs, the three most prominent genera were *Thauera* at 1.93%, *Pseudomonas* at 1.32%, and *Streptomyces* at 1.32%. Conversely, in advanced biological WWTPs, the three most prominent genera were *Pseudomonas* at 1.44%, *Thauera* at 1.42%, and *Streptomyces* at 1.38%. The data indicated that both types of plants have similar microbial communities. Figure 9 compares the most common genera between different WWTPs. The most-detected genus was *Desulfobacter* (4.82%) in preliminary WWTPs, *Thauera* (1.93%) in biological WWTPs, *Pseudomonas* (1.44%) in advanced biological WWTPs, *Acidovorax* (1.85%) in biological package WWTPs, and *Pseudomonas* (11.55%) in plant-based (vegetation-based) WWTPs (Figure 9). No significant difference was found at the phylum or genus level when comparing data from advanced biological WWTPs and UV recovery units (*p* > 0.05).

### ARGs in DWTPs and WWTPs

No ARG markers were detected in water samples from raw water inlets and ozonation unit outlets from DWTPs. Supplementary Table 3 presents the identified ARG markers in all samples collected from WWTPs. ARG markers were detected in both the inlet and outlet samples of preliminary WWTPs indicating that these facilities did not hinder the spread of the markers.

Different ARG markers were found in the inlet samples of biological, advanced biological, and plant-based WWTPs; these gene markers were absent in the outlet samples. This suggests that these facilities limited the spread of ARG markers. However, biological package WWTPs were found to limit the spread of ARG markers to a limited extent. No ARG markers were detected in the inlet and outlet samples of membrane wastewater recovery plants nor in the UV recovery facility sample.

The *ANT(3″)*, *Erm*, and *Sul* resistance gene markers were detected in all raw WWTPs samples (Table 2). These genes are associated with resistance to aminoglycosides, erythromycin, and sulfonamide antibiotics, respectively. Results indicated that various WWTPs exhibit differing profiles of antimicrobial resistance gene markers, with some plants detecting multiple genes in raw wastewater but showing no detection in treated wastewater, while others have a limited number of genes present in both raw and treated samples. This varies depending on the efficiency of the treatment processes and the design of the plants (Table 2).

**Table 2:** Distribution of detected antimicrobial resistance gene markers in different wastewater treatment plants.

WWTPs	Raw Wastewater-Inlet	Treated Wastewater-Outlet
Preliminary WWTPs	*AAC (6′); ANT (3″); APH (3″); APH (6); Ere; Erm; LNU; Sul; OXA*	*ANT (3″); APH (3″); Ere; Erm; LNU, OXA; Sul*
Biological WWTPs	*ANT (3″); Ere; Erm; LNU, OXA, Sul*	Not Detected
Advanced Biological WWTPs	*AAC (6′); ANT (3″); APH (3″); APH (6); Ere; Erm; LNU, OXA; Sul; Tet*	Not Detected
Biological Package WWTPs	*ANT (3″); APH (6); Ere; Erm; LNU; OXA, Sul*	*Sul*
Plant-based WWTPs	*ANT (3″)*	Not Detected

## Discussion

Current surveillance systems cannot adequately prevent, control, or intervene in infectious diseases, but new molecular detection methods have led to the development of water-based surveillance systems. These novel epidemiological tools could be used for early detection of outbreaks. This new approach assumes that both drinking water and wastewater monitoring can track newly emerging and reemerging infectious diseases at the community level in a comprehensive and real time way. It can further prevent the outbreaks caused by these microorganisms (Aborode et al., 2021; Sims and Kasprzyk-Hordern, 2020; Bruno et al., 2022). This study reports microbial metagenomic analyses of DWTP and WWTP waters under ISKI to obtain genomic epidemiology results related to these facilities.

### Metagenomic analysis of DWTPs

Bruno et al. (2022) examined 231 studies on microbiome analysis in DWTPs and delineated the foundational microbiome profile in these facilities. They highlighted that different disinfection or filtration practices could make it challenging to identify a preserved core taxonomic group. Nonetheless, they reported a core microbiome comprising Proteobacteria, Nitrospirae, Planctomycetes, Acidobacteria, Bacteroidetes, and Chloroflexi in the waters of these treatment plants. Brindefalk et al. (2022) studied 200 raw water sources in Sweden and reported that Actinobacteria, Bacteroidetes, and Proteobacteria constituted approximately 80–90% of all phyla. They found a slightly higher prevalence of Actinobacteria in samples from northern Sweden and a significant presence of Chloroflexi in central Sweden.

Despite the high diversity of bacterial communities in water, Proteobacteria dominate the bacterial community in drinking water. With current regulations, water from drinking water facilities should cause less than 1 infection per 10,000 people annually (Brindefalk et al., 2022; Mahajna et al., 2022). However, both microbial contaminants and environmental pathogens can potentially re-contaminate this water. Various pathogens like *Pseudomonas aeruginosa* can survive in drinking-water distribution systems despite low nutrient concentrations (Mahajna et al., 2022). Our study also identified that both raw drinking water and raw water examined after ozonation predominantly contained Proteobacteria.

The importance of ozone treatment is emphasized in many drinking water facilities, but there is limited data on its impact on microbial composition (Li et al., 2021). Li et al. reported that changes in bacterial diversity and network structure after ozone treatment were not solely due to ozone treatment—particularly as they occurred concurrently with an increase in temperature. Maguvu et al. (2020) reported a dominance of Proteobacteria and Planctomycetes in raw water from drinking water facilities. When ozone and chlorination were applied concurrently, they noted high levels of Proteobacteria and Firmicutes, respectively.

In our study, Proteobacteria remained predominant despite a decrease after ozone treatment compared to raw inlet waters. This led us to consider the necessity of longer-term monitoring including the need to consider temperature fluctuations. The analysis of raw waters from DWTPs and samples taken from the outlet of ozone units did not yield any statistically significant results from the phylum level to the genus level (*p* > 0.05). At the species level, however, statistically significant differences were observed in *Novosphingobium* sp. P6W (*p* = 0.003), *M. mesophilicum* (*p* = 0.02), *M. mesophilicum* SR1.6/6 (*p* = 0.02), and *Mesorhizobium* sp. 8 (*p* = 0.04). Statistically significant increases were detected in the quantities of these species after ozone treatment (*p* < 0.05). *Methylobacterium* can lead to pseudo-outbreaks based on water sources in dental clinics; this strain is known to be resistant to chlorination (Rice et al., 2000).

*Novosphingobium* sp. P6W can have diverse natural sources. *Novosphingobium* comprises Gram-negative bacteria that are commonly found in the environment and can be present in soil, in water systems, on plant roots, and in the intestines of some animals. They can also exhibit resistance to industrial pollutants—especially aromatic compounds (Wang et al., 2018). *Mesorhizobium* bacteria are typically involved in symbiotic relationships in the soil. These bacteria reside in root nodules of plants, particularly legumes (such as beans, peas, clover, etc.), which offer nitrogen fixation to the plant. *Mesorhizobium* can also be found in a free-living form and may be widely distributed in the soil (Lindström and Mousavi, 2020). Further information is needed on how the significant species in the analyses enter, spread, or pose potential risks in relevant facilities. Additional research and analysis are necessary to identify the sources of these species and understand the factors that could impact the quality of water treatment systems.

### Metagenomic analysis of WWTPs

Among the entrance and exit samples of WWTPs, there was a statistically significant decrease (*p* = 0.049) in the fungal order Hypocreales and an increase (*p* = 0.049) in the bacterial family Christensenellaceae (*p* < 0.05). The composition of the Christensenellaceae family can vary in fecal samples from different geographical regions and is particularly associated with the human gut microbiome. It is known to be associated with both metabolic diseases and the risk of colorectal cancer. The family has also been reported to trigger host genes associated with colorectal cancer (Waters and Ley, 2019). The use of recycled wastewater in agricultural areas, such as agricultural irrigation, can lead to serious problems due to fecal contamination (Khalid et al., 2018). The fungal order Hypocreales mostly comprises pathogenic genera such as *Fusarium* and *Trichoderma* and is one of the most commonly detected orders in WWTPs (Buratti et al., 2022).

De Celis et al. (2020) examined the bacterial community dynamics of an advanced biological WWTP with membrane bioreactors in a municipality in Spain through 16S rRNA sequencing. The main phyla detected in the wastewater were Proteobacteria, Bacteroidetes, Chloroflexi, Planctomycetes, and Actinobacteria. There were more Proteobacteria in winter and spring samples. Other phyla like Bacteroidetes, Chloroflexi, and Planctomycetes were less frequently detected in these months indicating that even significant phyla exhibited seasonal variations.

Begmatov et al. (2022) reported that in advanced biological WWTPs in Moscow, the dominant bacterial communities were composed of Proteobacteria (27.8%) followed by Bacteroidota (15.7%), Actinobacteriota (12.5%), Chloroflexi (6.6%), Myxococcota (5.9%), Firmicutes (5.6%), Patescibacteria (5.5%), Verrucomicrobiota (4.5%), Bdellovibrionota (3.9%), Nitrospirota (2.7%), and Planctomycetota (1.3%). They emphasized that such studies are crucial in determining the variations in microbial composition within the plants and understanding the functioning of microorganisms. Kim et al. (2019) reported that Proteobacteria was the dominant phylum in biological WWTPs in South Korea and Vietnam, constituting 40 to 60% of the total active bacteria. They indicated that other significant phyla included Bacteroidetes (20–30%), Firmicutes (5–10%), and Actinobacteria (3–8%). They believed that Proteobacteria play a role in the removal of organic pollutants like nitrogen, phosphorus, and aromatic compounds.

In our study, the higher presence of the Proteobacteria in preliminary WWTPs compared to biological WWTPs suggests better adaptation of this phylum to sewage containing coarse particles. The Proteobacteria phylum includes bacteria that play significant roles in wastewater treatment processes such as nitrification, denitrification, sulfate reduction, and methane oxidation. The higher presence of the phylum Acidobacteria in biological WWTPs compared to preliminary WWTPs indicates better adaptation of this phylum to wastewater with high organic content and neutral or alkaline pH levels. Acidobacteria are bacteria that contribute to the carbon cycle by breaking down organic matter.

The higher presence of the genus *Myxococcus* in biological WWTPs compared to preliminary treatment plants indicate that this genus comprises bacteria that break down organic matter aerobically to produce energy. *Myxococcus* may also contribute to biofilm formation through polysaccharide production. Similarly, Zhang et al. (2023) also detected *Myxococcota* (6.5 ± 1.3%).

Furthermore, the higher presence of the genus *Rothia* in preliminary WWTPs compared to biological treatment plants suggests that this genus includes pathogenic bacteria sourced from humans and animals. *Rothia* can also lower pH by degrading organic matter and producing acid (Chen et al., 2018; Miyashita, 2015). The phylum Verrucomicrobia comprises microorganisms with various metabolic functions, such as organic matter degradation, nitrogen transformation, and phosphorus uptake (van Teeseling et al., 2013). *Akkermansia muciniphila* is a probiotic member of this phylum that maintains gut health by digesting the mucus layer and providing protection against obesity (Zhang et al., 2019).

Our data appear consistent with the literature. Many genera identified at higher levels in preliminary WWTPs compared to advanced biological treatment plants are soilborne bacteria capable of establishing symbiotic relationships with plant roots. The increased presence of these bacteria in preliminary WWTPs might result from the higher content of soil particles originating from agricultural or green areas in the wastewater entering these treatment plants. Some of these bacteria can perform nitrogen fixation and utilize nitrogen-containing compounds, which potentially contributes to the higher nitrogen concentration observed in preliminary WWTPs.

However, more detailed data are needed to assess the performance of treatment processes. The different processes used in treatment plants and seasonal variations may impact microbial composition. This necessitates further research to determine how these differences affect treatment efficiency and how to best establish the fundamental microbiome.

### ARGs in WWTPs

Hultman et al. (2018) investigated the formation of ARGs in preliminary WWTPs (with advanced wastewater treatment) in Finland. Similar to our study, their research revealed various ARGs in the wastewater of these facilities including genes conferring resistance to antibiotics like tetracycline, erythromycin, and sulfonamides. The researchers also reported that ARGs were more common in sludge from facilities with a high influx of wastewater.

Despite numerous studies worldwide focusing on ARGs in samples from WWTPs, no study has yet been conducted on ISKI-affiliated facilities until this report. Studies conducted in other countries have identified different ARGs in various facilities suggesting that wastewater is a significant source of overall dissemination of these genes. WWTPs are considered essential tools in safeguarding against these genes (Pazda et al., 2019; Sambaza and Naicker, 2023). In biological processes, the removal of ARGs depends on factors such as the structure and function of the microbial community, oxygen concentration, efficiency of nutrient removal, sludge retention time, and incubation time in water (Pazda et al., 2019; Sambaza and Naicker, 2023). In advanced biological processes, ARGs can be removed through chemical processes such as ozonation, chlorination, Fenton oxidation, and other advanced oxidation processes—as well as physicochemical processes like UV radiation and ionizing radiation.

In phytobiological WWTPs, plants remove ARGs through the secretion of antimicrobial compounds by root systems or via the adsorption of bacteria carrying the genes. Establishing and maintaining an appropriate microbial community structure is crucial for ARG removal in biological WWTP processes. To achieve this, understanding the fundamental microbial structure is essential, and parameters such as oxygen concentration, nutrient removal efficiency, sludge retention time, and incubation time in water should be optimized to shape this structure. In advanced biological WWTP processes, chemical or physicochemical methods can be used for ARG removal. However, the disadvantages associated with these processes should also be considered: cost, energy consumption, and byproduct formation.

In phytobiological WWTPs, the selection of plant species and root systems is crucial for ARG removal. Plants can prevent the spread of ARGs by secreting antimicrobial compounds and adsorbing bacteria carrying ARGs. However, plants also have the potential to release ARGs into the environment (Mutuku et al., 2022; Sambaza and Naicker, 2023; Wang et al., 2020).

Our study lacked the determination of output values from DWTPs, including chlorination, which limits our knowledge regarding the current status of these bacterial species. Furthermore, the microbial metagenomic analysis method used here can only provide information about the genomic structure of microbial profiles in the examined samples without determining whether this genomic structure belongs to a living or dead microorganism, which is another limitation of the study. More details and data are required to assess the performance of treatment processes adequately. Different processes employed in WWTPs can impact the microbial composition, and longer and more extensive studies are needed to understand how these differences affect treatment efficiency. However, our study has value in providing essential genomic epidemiological data for both the fundamental microbiomes and public health in a large city like Istanbul. The majority of the sequencing reads were left unidentified even though we used one of the most recent sets of publicly accessible nucleotide sequences, which includes references from Archaea, bacteria, viruses, plasmids, protozoa, fungi, and humans. We hypothesize that unmapped reads may be the result of sequencing errors or that they may belong to unknown prokaryotes. A high proportion of unmapped reads in microbiome studies emphasizes the importance of meta-analysis and other strategies for confirming earlier research.

## Conclusion

This study presents initial microbial profiling data from input and output samples of both drinking water and wastewater facilities operated under ISKI, which serves the large city of Istanbul. We found no significant microbial risk differentiation between biological WWTPs and advanced biological WWTPs. We aimed to gather specific data for certain wastewater and drinking water basins, but comprehensive analyses were hindered by the complexity of multiple basins feeding water sources and transmitting wastewater to treatment plants; the need for retrospective analysis; and the limited number and frequency of samples. However, these data can serve as preliminary information for future research.

More comprehensive studies involving multi-sample tracking in these facilities and the water basins that feed them are critically important. Particularly, these studies could reveal seasonal changes within the facilities and microbial profile differences among the water basins, thus providing an opportunity for comparisons with the current data. There are currently no regulations providing standards for microbial metagenomic data, and developing such standards would facilitate the establishment of more effective active monitoring systems using these methods. This would allow for better assessment of the contributions of these facilities to public health in major cities like Istanbul. The monitoring of these fundamental microbial profile data using standardized methods could enable more rapid actions against microbial risks.

## Data Availability

Everyone can access the dataset using ArrayExpress with Accession number: E-MTAB-14353. 514 Link: https://www.ebi.ac.uk/biostudies/arrayexpress/studies/E-MTAB-14353?key=8bb81ad7-3f2f51544fc-99c8-995992b0b535.
