# Supplementary material for: Comparative microbial metagenomic analysis of drinking water plants and wastewater treatment plants in Istanbul
Source: Front Microbiol. 2025 Jan 20;16:1488268. doi: 10.3389/fmicb.2025.1488268 (PMC11788275; doi:10.3389/fmicb.2025.1488268)

Supplemantery Tables

Table 1. Information about DWTPs where samples of raw water and from the ozonation unit exit were collected.

DRINKING WATER PLANT (DWTP)	SAMPLE TYPE (Sample Taking Point)	DATE	DWTP CAPACITY (m^3/day)	TOTAL AREA (m^2)
Cumhuriyet Drinking Water Treatment Plant	Raw Water- Inlet	31.10.2022	720.000	3.710.628
Büyükçekmece Drinking Water Treatment Plant	Raw Water- Inlet	14.11.2022	400.000	250 000
Taşoluk Drinking Water Treatment Plant	Raw Water- Inlet	29.11.2022	100.000	92.000
Elmalı Drinking Water Treatment Plant	Raw Water- Inlet	31.10.2022	40.000	63.450
Silivri Hallaçlı Drinking Water Treatment Plant	Raw Water- Inlet	14.11.2022	22.000	3.150
Şile Ağva Drinking Water Treatment Plant	Raw Water- Inlet	07.11.2022	17.000	2.000
Ömerli Emirli I ve Emirli II Drinking Water Treatment Plant (Common Inlet)	Raw Water- Inlet	06.12.2022	500.000	36.000
Ömerli Drinking Water Treatment Plant Raw Water 1 (Orhaniye, Osmaniye, Muradiye)	Raw Water- Inlet	06.12.2022	300.000	13.000
Kağıthane Terkos Drinking Water Treatment Plant	Raw Water- Inlet	28.11.2022	300.000	270.000
Kağıthane Alibey Drinking Water Treatment Plant	Raw Water- Inlet	28.11.2022	300.000	270.000
İkitelli Terkos Drinking Water Treatment Plant	Raw Water- Inlet	21.11.2022	400.000	240.000
İkitelli Sazlıdere Drinking Water Treatment Plant	Raw Water- Inlet	21.11.2022	400.000	240.000
Ömerli Barajı Sal Pompa Terfi Merkezi	Raw Water- Inlet	06.12.2022	300.000	4.200
Ömerli Emirli I ve Emirli II Drinking Water Treatment Plant Ozonation Unit	Ozonation Unit- Outlet	06.12.2022	500.000	36.000
İkitelli Drinking Water Treatment Plant	Ozonation Unit- Outlet	21.11.2022	400.000	240.000
Kağıthane Drinking Water Treatment Plant	Ozonation Unit- Outlet	28.11.2022	300.000	270.000
Ömerli Drinking Water Treatment Plant Raw Water 1 (Orhaniye, Osmaniye, Muradiye) Ozonation Unit	Ozonation Unit- Outlet	06.12.2022	300.000	13.000

Elmalı Drinking Water Treatment Plant	Ozonation Unit- Outlet	31.10.2022	40.000	63.450

Table 2. Sampling information for WWTPs and sampling details related to these facilities.

WASTEWATER TREATMENT PLANT (WWTP)	SAMPLE TYPE (Sample Taking Point)	DATE	Flowrate (m^3/day)	Total Area (m^2)	Total Area of Basin (m^2)
Yenikapı Wastewater Pre-treatment Plant	Raw Wastewater - Inlet	29.11.2022	864.000	21.400	177.735.741
Yenikapı Wastewater Pre-treatment Plant	Treated Wastewater - Outlet	29.11.2022	864.000	21.400	177.735.741
Kadıköy Wastewater Pre-treatment Plant	Raw Wastewater - Inlet	11.11.2022	1.420.000	60.000	76.850.000
Kadıköy Wastewater Pre-treatment Plant	Treated Wastewater - Outlet	11.11.2022	1.420.000	60.000	76.850.000
Küçüksu Wastewater Pre-treatment Plant	Raw Wastewater - Inlet	02.11.2022	640.000	13.950	63.750.000
Küçüksu Wastewater Pre-treatment Plant	Treated Wastewater - Outlet	02.11.2022	640.000	13.950	63.750.000
Baltalimanı Wastewater Pre-treatment Plant	Raw Wastewater - Inlet	28.11.2022	N/A	269.800	71.120.400
Baltalimanı Wastewater Pre-treatment Plant	Treated Wastewater - Outlet	28.11.2022	N/A	269.800	71.120.400
Paşabahçe Wastewater Pre-treatment Plant	Raw Wastewater - Inlet	02.11.2022	570.000	33.312	28.300.000
Paşabahçe Wastewater Pre-treatment Plant	Treated Wastewater - Outlet	02.11.2022	570.000	33.312	28.300.000
Tuzla BWTP and Tuzla ABWTP	Raw Wastewater- Inlet (Tuzla BWTP and Tuzla ABWTP Raw Wastewater – Common Inlet)	11.11.2022	450.000	550.000	111.130.000
Tuzla Biological Wastewater Treatment Plant	Treated Wastewater - Outlet	11.11.2022	150.000	550.000	111.130.000
Gümüşyaka Biological Wastewater Treatment Plant	Raw Wastewater - Inlet	14.11.2022	4.400	15.548	4.884.600
Gümüşyaka Biological Wastewater Treatment Plant	Treated Wastewater - Outlet	14.11.2022	4.400	15.548	4.884.600
Ataköy Advanced Biological Wastewater Treatment Plant Stage 1	Raw Wastewater - Inlet	21.11.2022	360.000	400.000*	42.504.400
Ataköy Advanced Biological Wastewater Treatment Plant Stage 1	Treated Wastewater - Outlet	21.11.2022	360.000	400.000*	42.504.400
Ambarlı Advanced Biological Wastewater Treatment Plant	Raw Wastewater - Inlet	21.11.2022	400.000	230.862	121.260.000
Ambarlı Advanced Biological Wastewater Treatment Plant	Treated Wastewater - Outlet	21.11.2022	400.000	230.862	121.260.000

Tuzla Advanced Biological Wastewater Treatment Plant	Treated Wastewater - Outlet	11.11.2022	450.000	550.000	111.130.000
Paşaköy Advanced Biological Wastewater Treatment Plant	Raw Wastewater - Inlet	02.11.2022	500.000	507.000	107.330.000
Paşaköy Advanced Biological Wastewater Treatment Plant	Treated Wastewater - Outlet	02.11.2022	500.000	507.000	107.330.000
Büyükçekmece Advanced Biological Wastewater Treatment Plant	Raw Wastewater - Inlet	14.11.2022	401.059	135.600	64.211.000
Büyükçekmece Advanced Biological Wastewater Treatment Plant	Treated Wastewater - Outlet	14.11.2022	401.059	135.600	64.211.000
Ataköy Advanced Biological Wastewater Treatment Plant Stage 2	Raw Wastewater - Inlet	21.11.2022	240.000	400.000*	42.504.400
Ataköy Advanced Biological Wastewater Treatment Plant Stage 2	Treated Wastewater - Outlet	21.11.2022	240.000	400.000*	42.504.400
Ataköy Advanced Biological Wastewater Treatment Plant Recovery System	Microfiltration Unit- Outlet	21.11.2022	N/A	400.000*	42.504.400
Ambarlı Advanced Biological Wastewater Treatment Plant Recovery System	Active Carbon Unit- Inlet	21.11.2022	N/A	230.862	121.260.000
Ambarlı Advanced Biological Wastewater Treatment Plant Recovery System	Ultrafiltration Unit- Outlet	21.11.2022	N/A	230.862	121.260.000
Sırapınar Biological Package Wastewater Treatment Plant	Raw Wastewater - Inlet	18.11.2022	281	1.860	450
Sırapınar Biological Package Wastewater Treatment Plant	Treated Wastewater - Outlet	18.11.2022	281	1.860	450
Şile Oruçoğlu Vegetative Wastewater Treatment Plant	Raw Wastewater - Inlet	07.11.2022	100	3.475	3.540.700
Şile Oruçoğlu Vegetative Wastewater Treatment Plant	Treated Wastewater - Outlet	07.11.2022	100	3.475	3.540.700
Paşaköy Advanced Biological Wastewater Treatment Plant UV Recovery System	Filtration Unit - Inlet	02.11.2022	100.000	507.000	107.330.000
Paşaköy Advanced Biological Wastewater Treatment Plant UV Recovery System	UV Unit- Outlet	02.11.2022	100.000	507.000	107.330.000

Table 3. Distribution of detected antimicrobial resistance gene markers in all wastewater samples

WASTEWATER TREATMENT PLANT	DETECTED ANTIMICROBIAL RESISTANCE GENES	SAMPLE TAKING POINT
Yenikapı Preliminary Wastewater treatment Plant	ANT (3''); Ere; Erm; LNU; Sul; Tet	Raw Wastewater- Inlet
Yenikapı Preliminary Wastewater treatment Plant	Erm; LNU, OXA	Treated Wastewater- Outlet
Kadıköy Preliminary Wastewater treatment Plant	ANT (3''); Ere; LNU; MFS-type; OXA; Sul	Raw Wastewater- Inlet
Kadıköy Preliminary Wastewater treatment Plant	AAC (6')-Ib-cr; ANT (3''); Ere LNU; Sul	Treated Wastewater- Outlet
Küçüksu Preliminary Wastewater treatment Plant	ABC-F; ANT (3''); APH (3''); Erm; MPH; Sul	Raw Wastewater- Inlet
Küçüksu Preliminary Wastewater treatment Plant	ANT (3''); APH (3''); Ere Erm; LNU, MPH; OXA, Sul	Treated Wastewater- Outlet
Baltalimanı Preliminary Wastewater treatment Plant	ANT (3''); APH (3''); APH (6); Ere Erm; LNU, OXA; Sul	Raw Wastewater- Inlet
Baltalimanı Preliminary Wastewater treatment Plant	AAC (6'); ANT (3''); APH (3''); Ere Erm; LNU, MPH; OXA, Sul	Treated Wastewater- Outlet
Paşabahçe Preliminary Wastewater treatment Plant	AAC (6'); ANT (3''); APH (3''); APH (6); Ere LNU; OXA, Sul	Raw Wastewater- Inlet
Paşabahçe Preliminary Wastewater treatment Plant	ANT (3''); APH (3''); APH (6); CARB, Ere; Erm; LNU; MPH; OXA; Sul	Treated Wastewater- Outlet
Tuzla BWTP and Tuzla ABWTP	Sul; TEM	Raw Wastewater- Inlet (Tuzla BWTP and Tuzla ABWTP Raw Wastewater – Common Inlet)
Tuzla Biological Wastewater Treatment Plant	-	Treated Wastewater- Outlet
Gümüşyaka Biological Wastewater Treatment Plant	ANT (3''); Ere Erm; LNU, MFS-type; OXA, Sul	Raw Wastewater- Inlet
Gümüşyaka Biological Wastewater Treatment Plant	-	Treated Wastewater- Outlet
Ataköy Advanced Biological Wastewater Treatment Plant Stage 1	ANT (3''); APH (3''); APH (6); Ere Erm; LNU, OXA; Sul; Tet	Raw Wastewater- Inlet
Ataköy Advanced Biological Wastewater Treatment Plant Stage 1	-	Treated Wastewater- Outlet
Ambarlı Advanced Biological Wastewater Treatment Plant	AAC (6'); ANT (3''); APH (3''); Ere Erm; LNU, MPH; OXA; Sul; Tet	Raw Wastewater- Inlet
Ambarlı Advanced Biological Wastewater Treatment Plant	-	Treated Wastewater- Outlet
Tuzla Advanced Biological Wastewater Treatment Plant	-	Treated Wastewater- Outlet
Paşaköy Advanced Biological Wastewater Treatment Plant	ANT (3''); APH (3''); APH (6); Ere Erm; GES, LNU; OXA, Sul	Raw Wastewater- Inlet
Paşaköy Advanced Biological Wastewater Treatment Plant	-	Treated Wastewater- Outlet
Büyükçekmece Advanced Biological Wastewater Treatment Plant	ANT (3''); APH (3''); Ere Erm; LNU, OXA; Sul; Tet	Raw Wastewater- Inlet
Büyükçekmece Advanced Biological Wastewater Treatment Plant	-	Treated Wastewater- Outlet
Ataköy Advanced Biological Wastewater Treatment Plant Stage 2	AAC (6'); ANT (3''); ANT (6); APH (3''); APH (6); CfxA; Ere; Erm; GES; LNU; MPH; OXA; Sul	Raw Wastewater- Inlet
Ataköy Advanced Biological Wastewater Treatment Plant Stage 2	-	Treated Wastewater- Outlet
Ataköy Advanced Biological Wastewater Treatment Plant Recovery System	-	Microfiltration Unit-Outlet
Ambarlı Advanced Biological Wastewater Treatment Plant Recovery System	-	Active Carbon Unit-Inlet
Ambarlı Advanced Biological Wastewater Treatment Plant Recovery System	-	Ultrafiltration Unit- Outlet
Sırapınar Biological Package Wastewater Treatment Plant	ANT (3''); APH (6); Ere; Erm; LNU; OXA, Sul	Raw Wastewater- Inlet
Sırapınar Biological Package Wastewater Treatment Plant	MFS-type; Sul	Treated Wastewater- Outlet
Şile Oruçoğlu Plant-Based Wastewater Treatment Plant	ANT (3'')	Raw Wastewater- Inlet
Şile Oruçoğlu Plant-Based Wastewater Treatment Plant	-	Treated Wastewater- Outlet
Paşaköy Advanced Biological Wastewater Treatment Plant UV Recovery System	Erm; Sul	Filtration Unit- Inlet
Paşaköy Advanced Biological Wastewater Treatment Plant UV Recovery System	-	UV Unit- Outlet

WWTP - Advanced

52-51

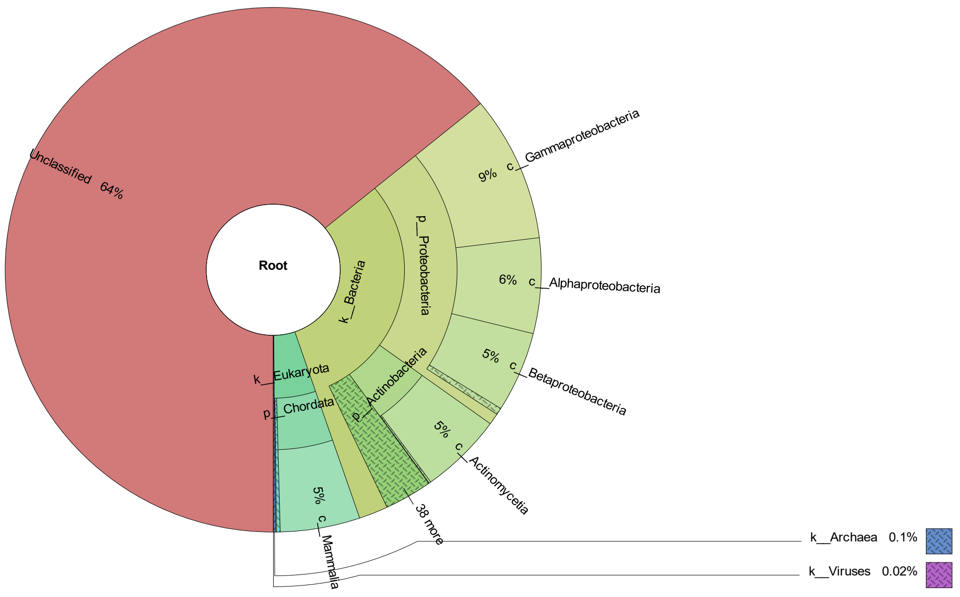

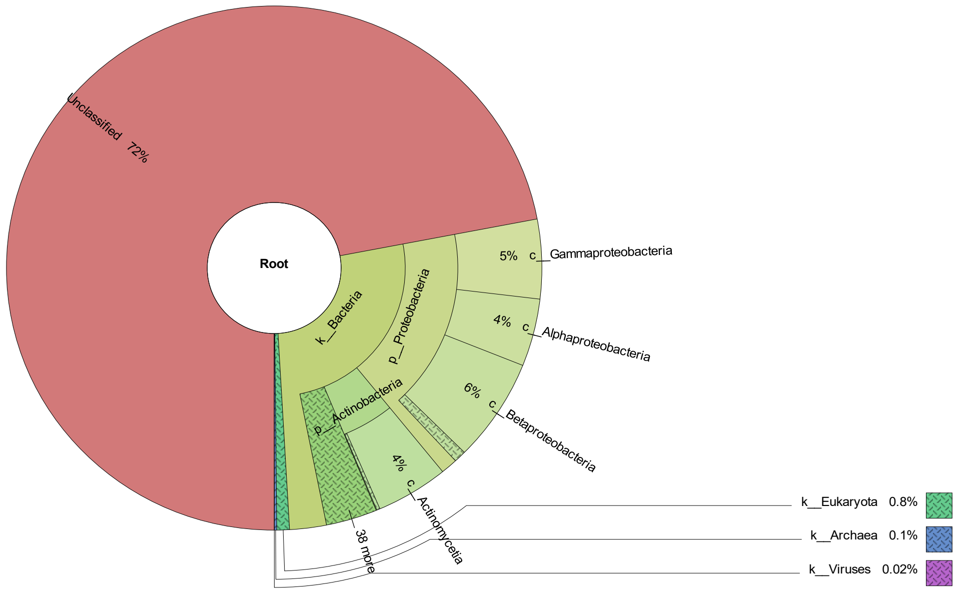

WWTP - Plant-based
50-49

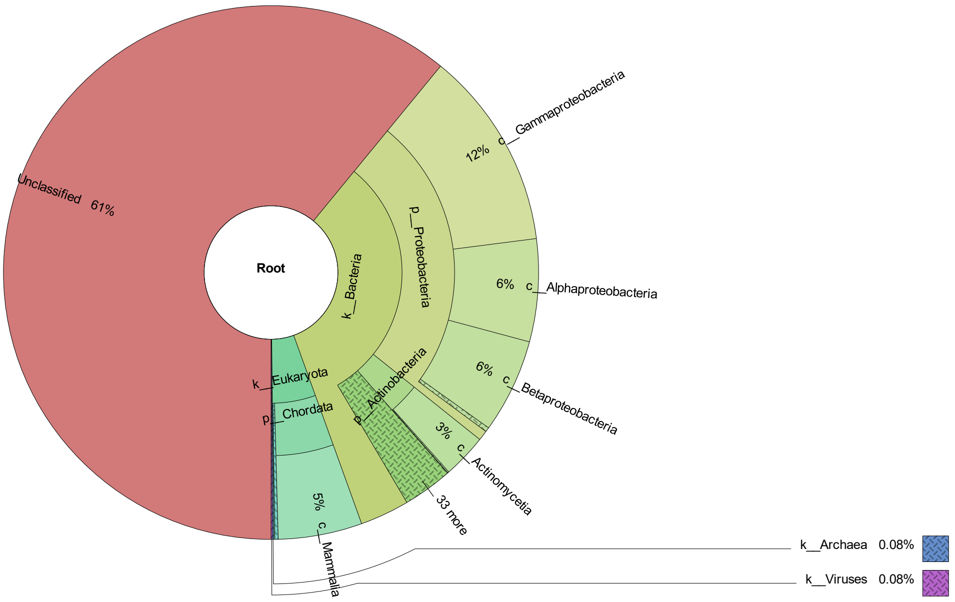

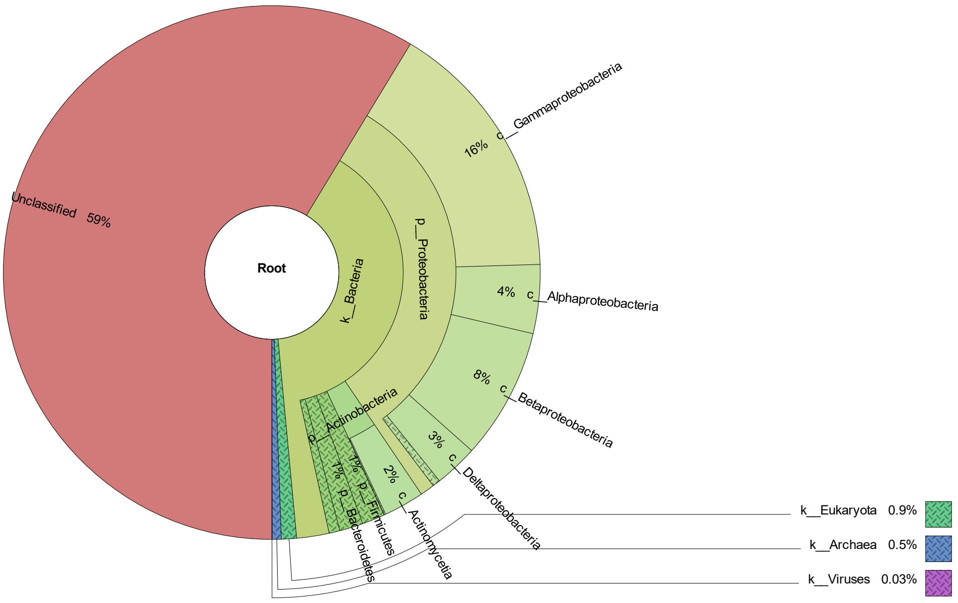

WWTP - Biological package
48-47

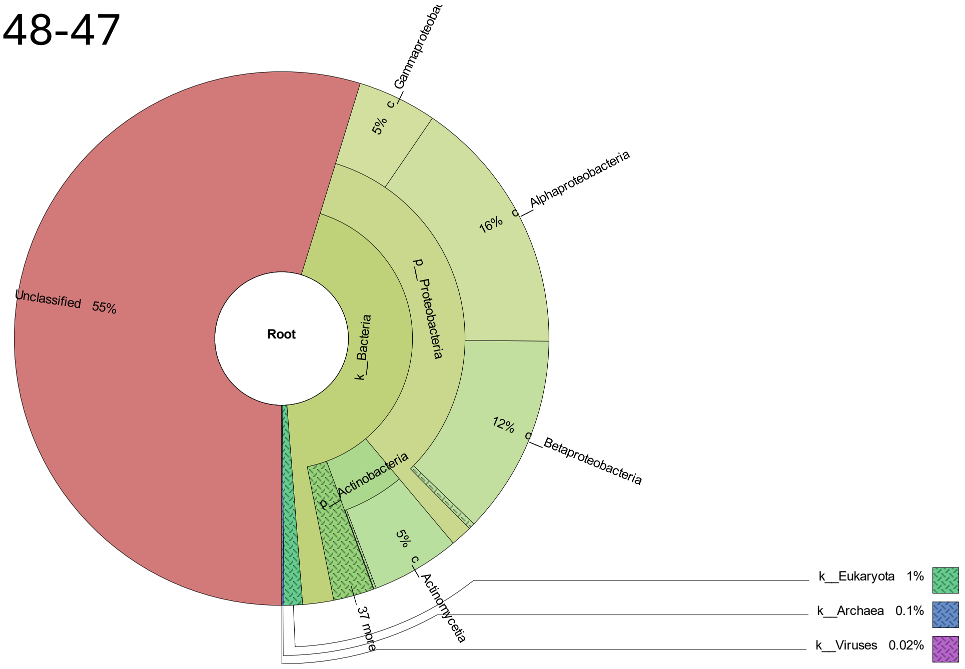

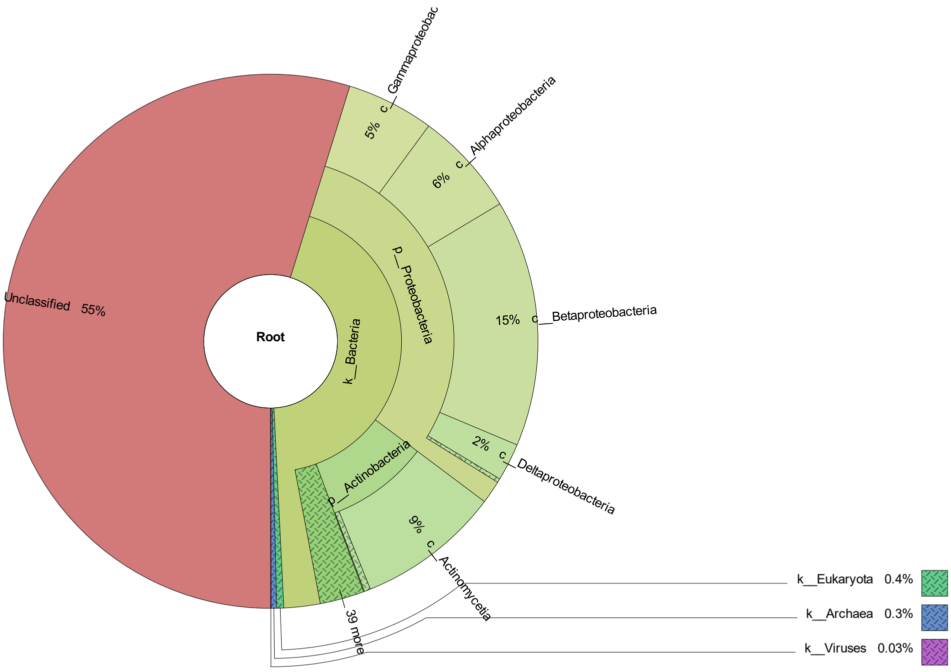

WWTP - Advanced
46-43

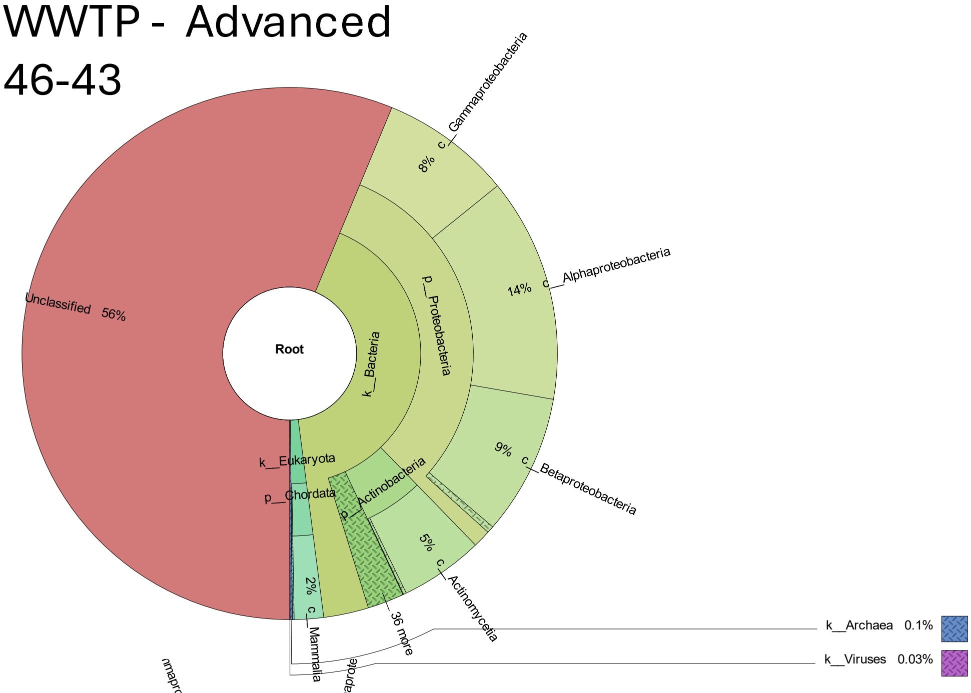

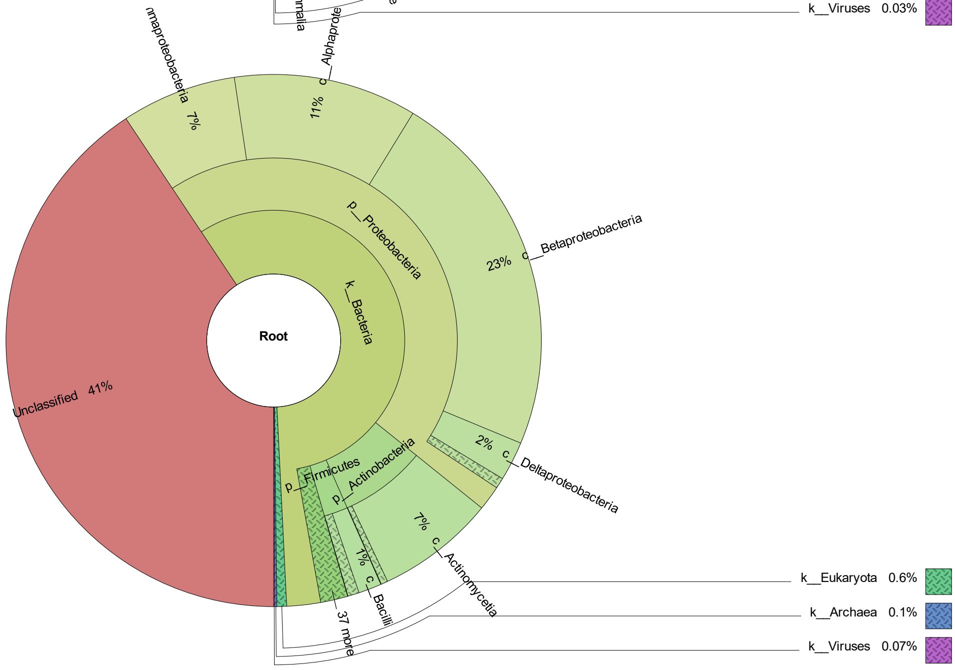

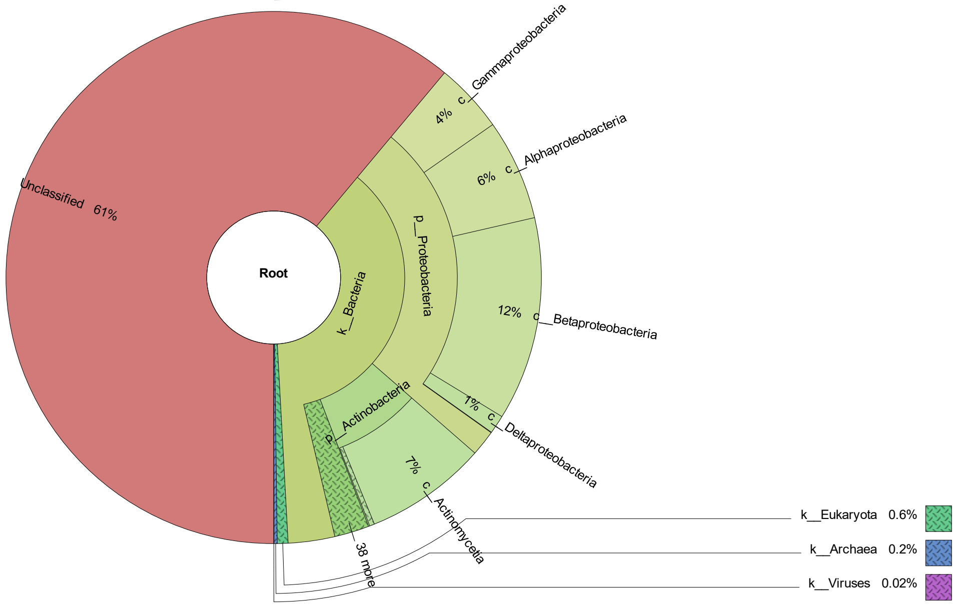

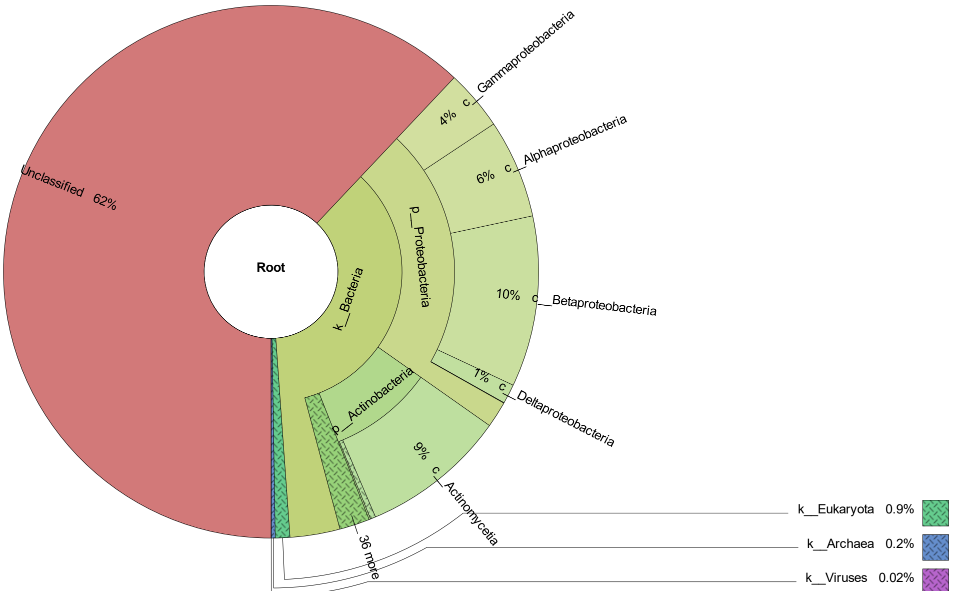

WWTP - Advanced
42-39

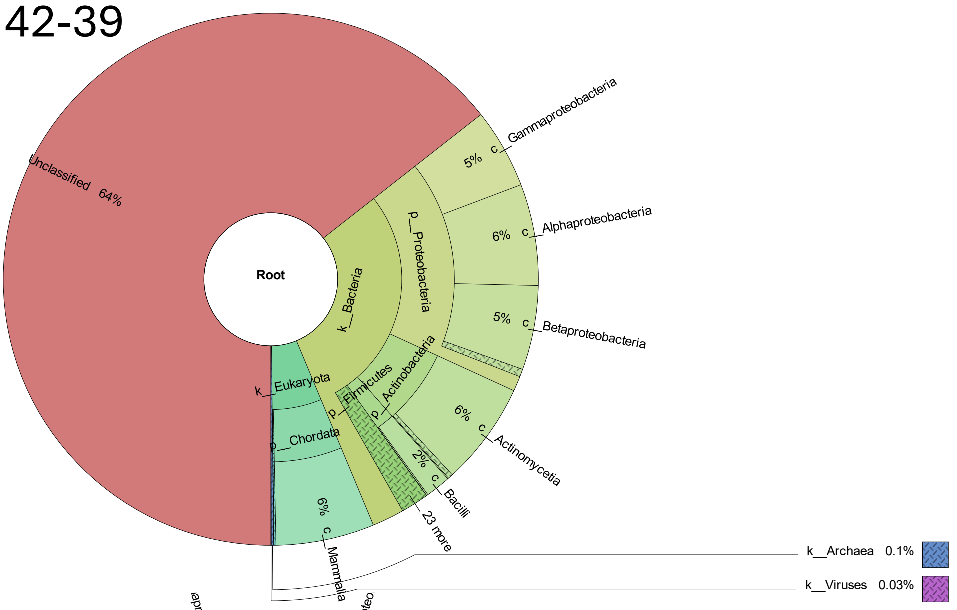

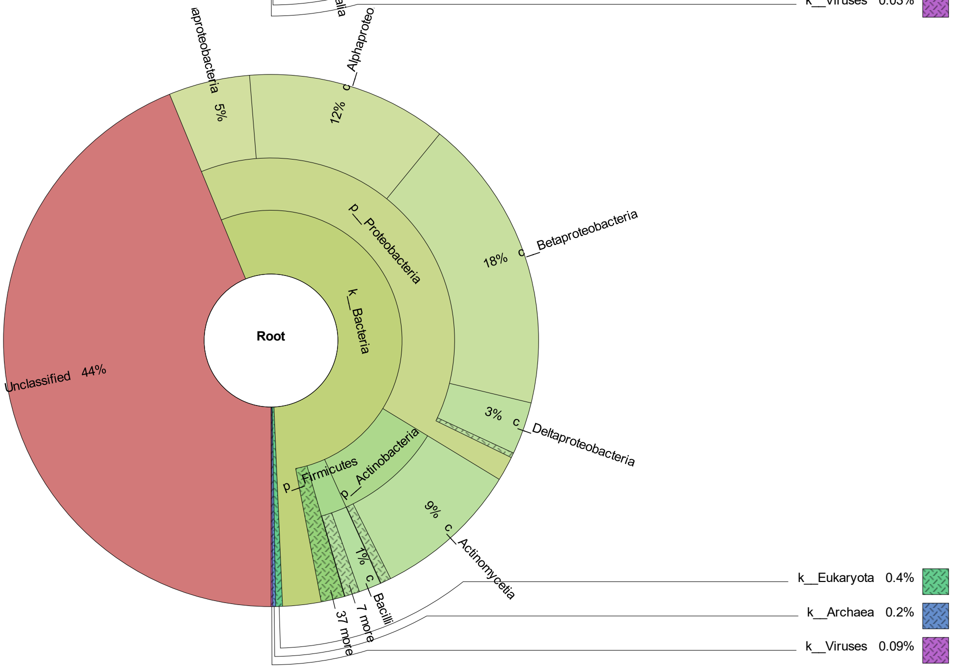

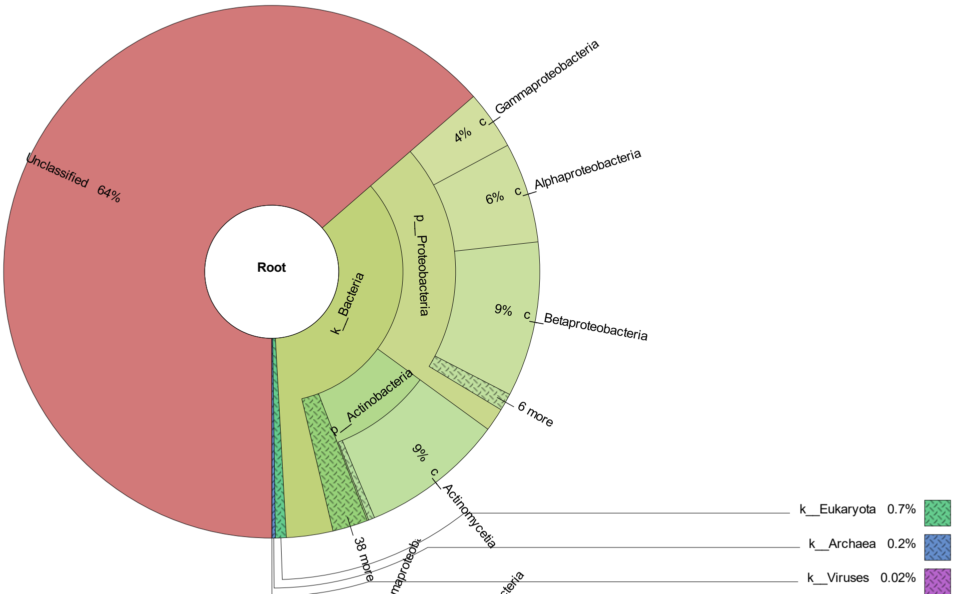

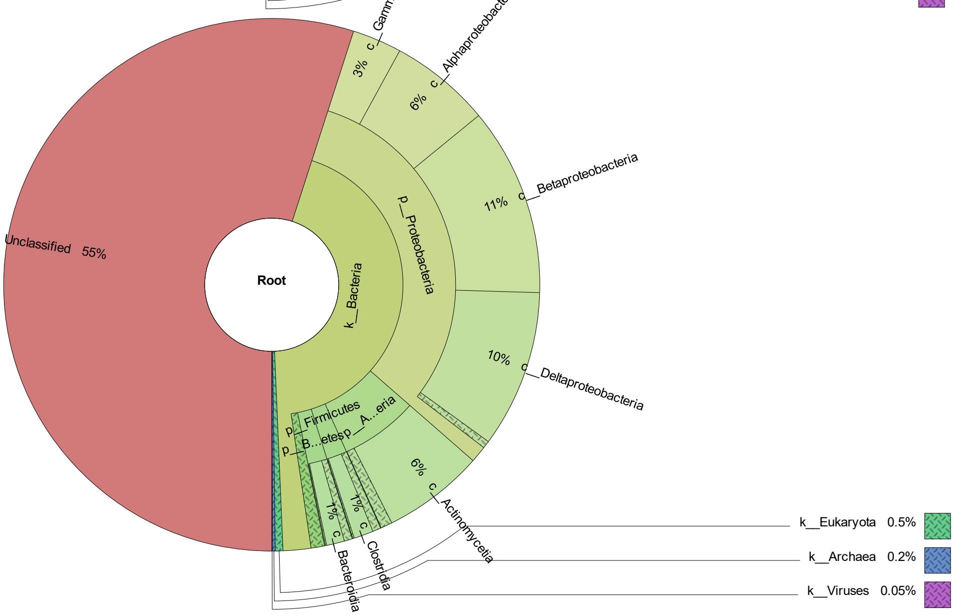

WWTP - Advanced
38-35

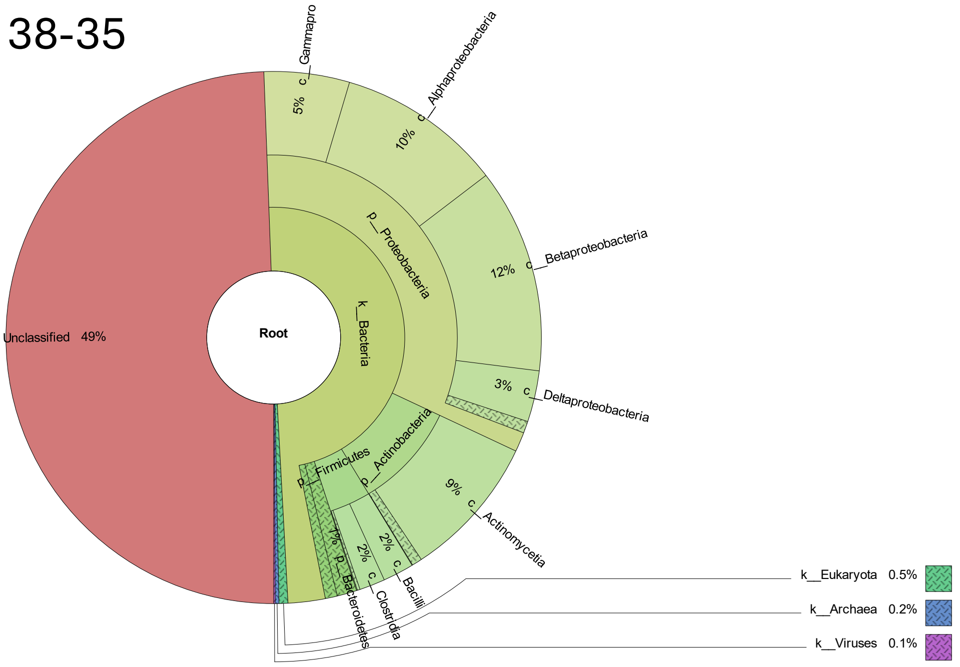

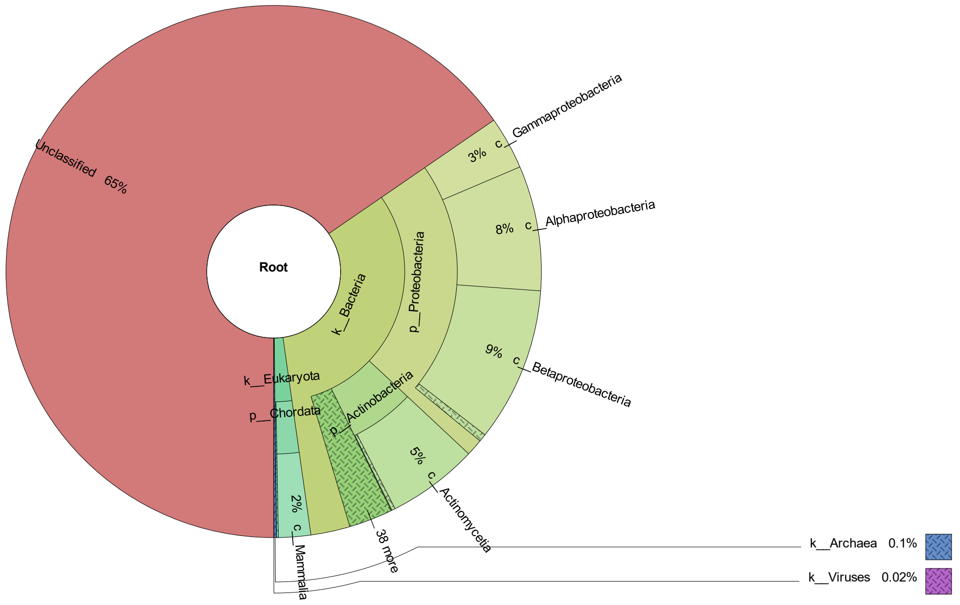

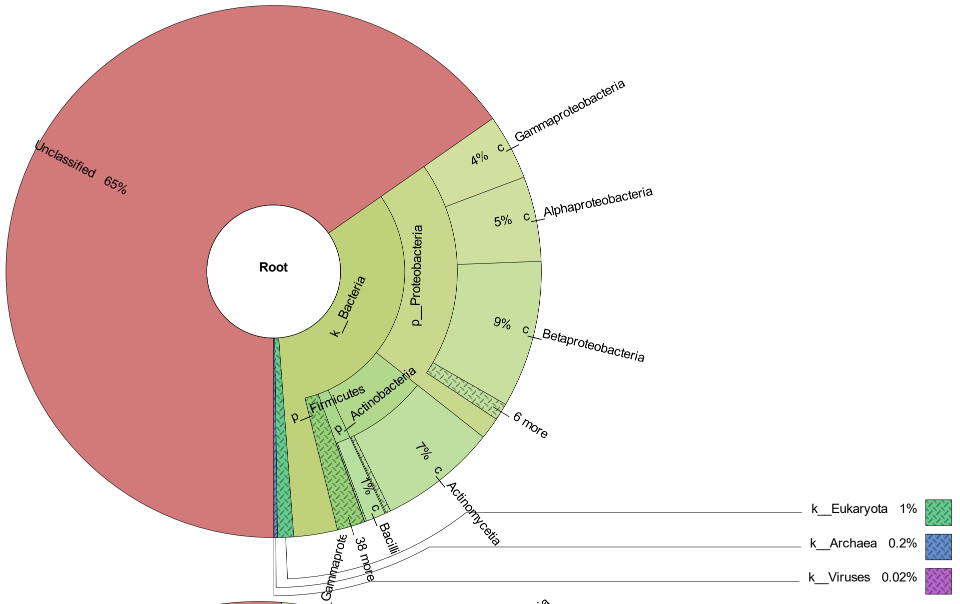

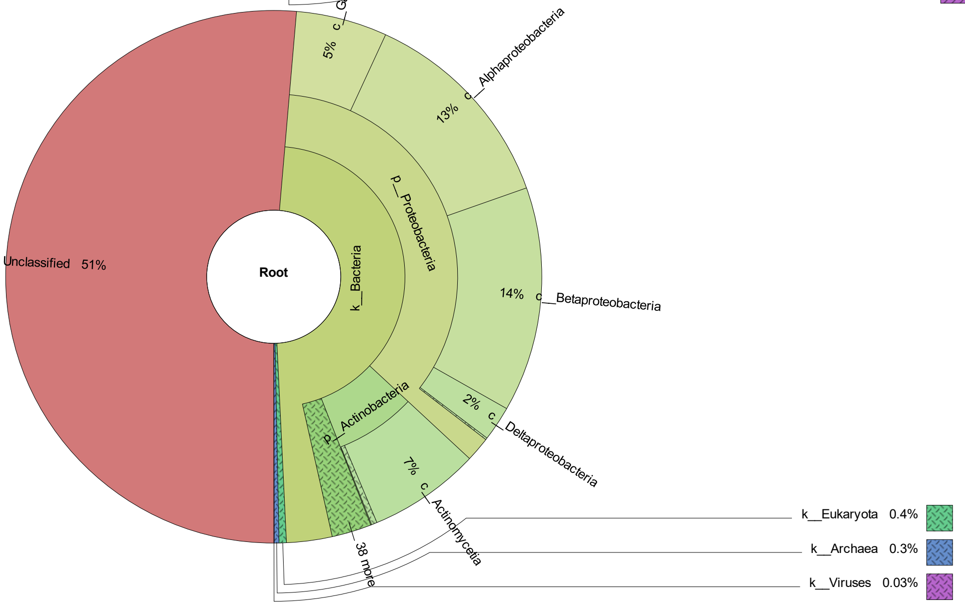

WWTP - Advanced
34-33

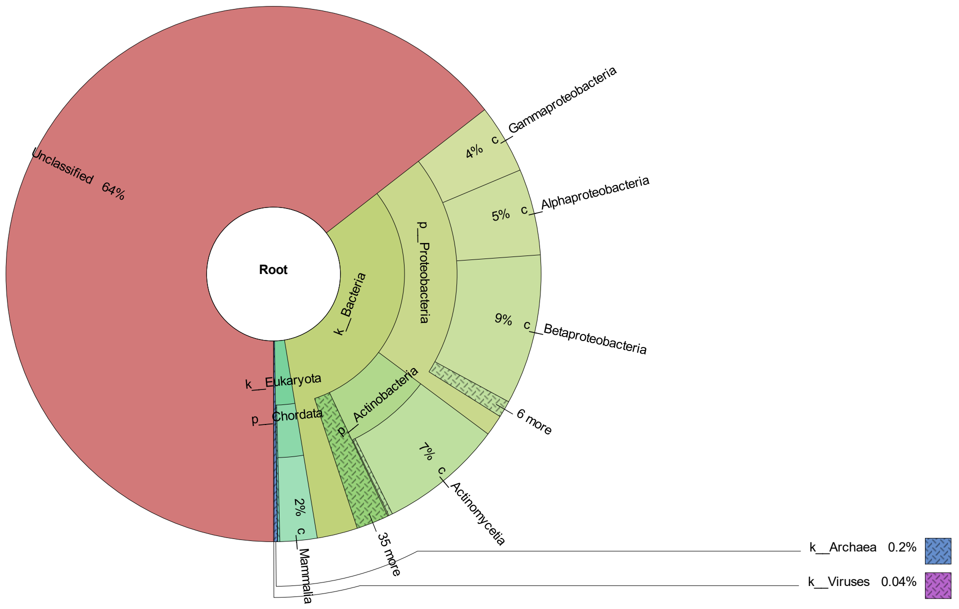

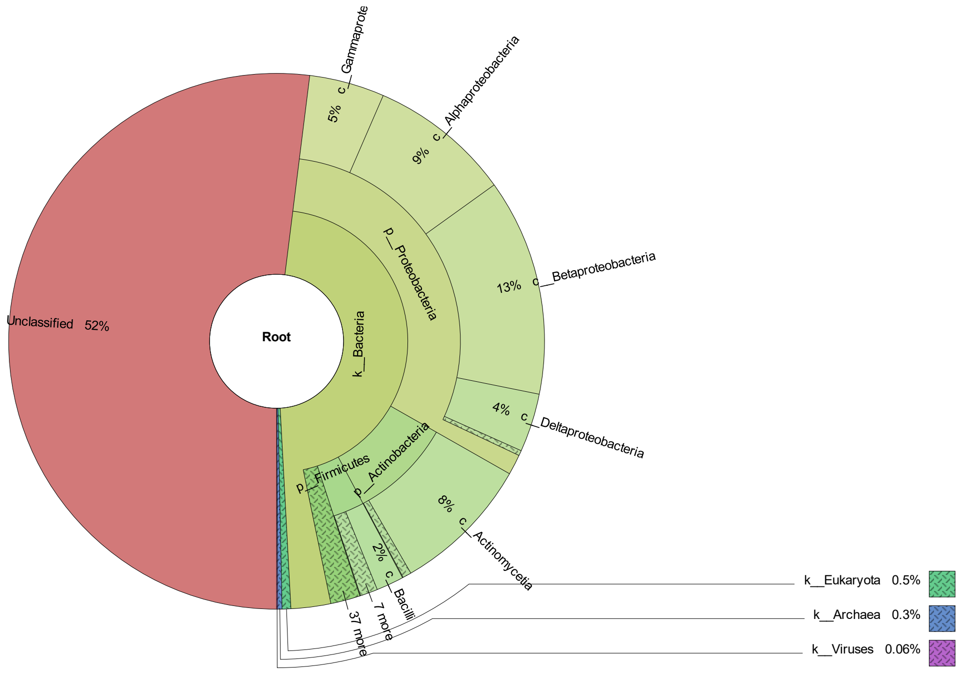

WWTP - Biological
32-29

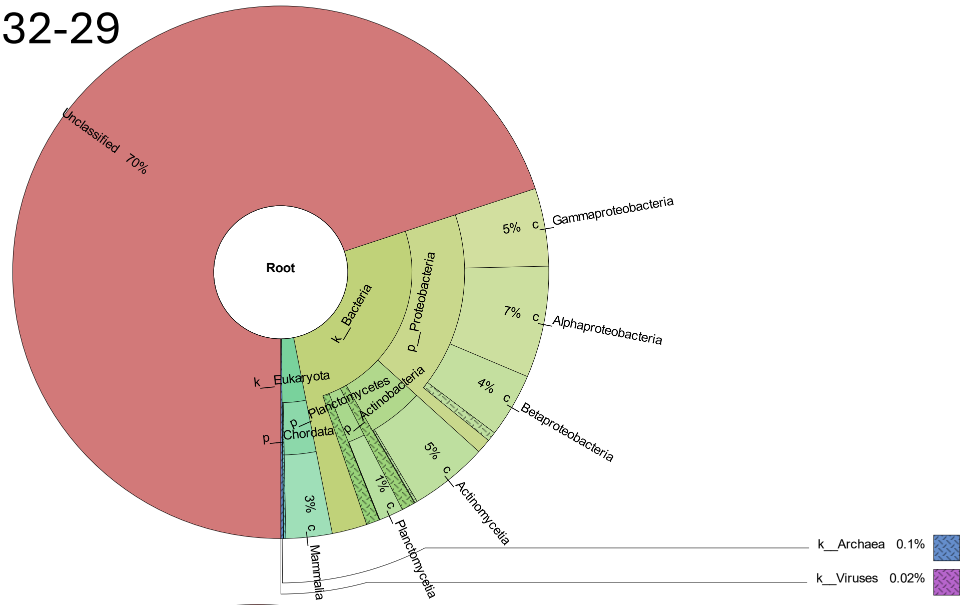

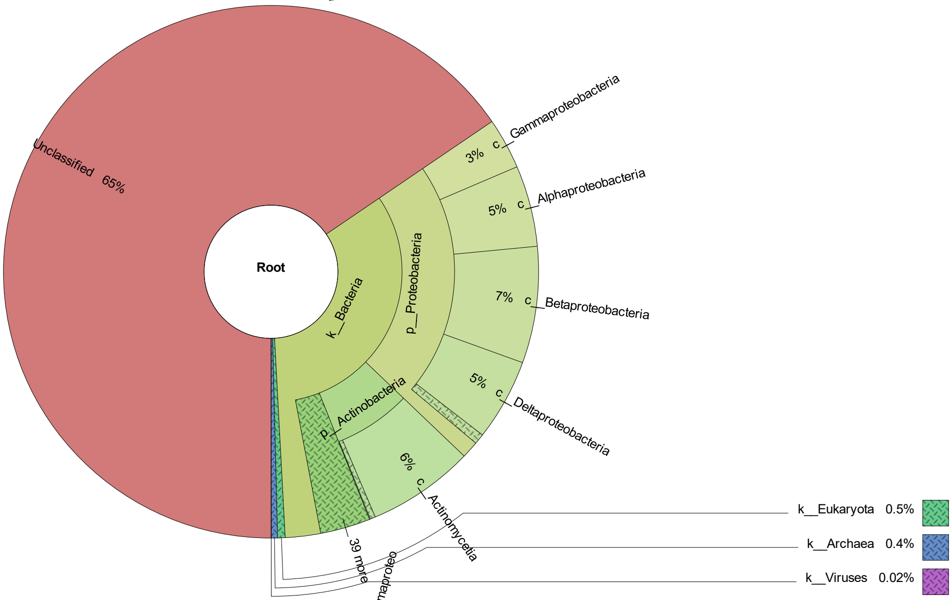

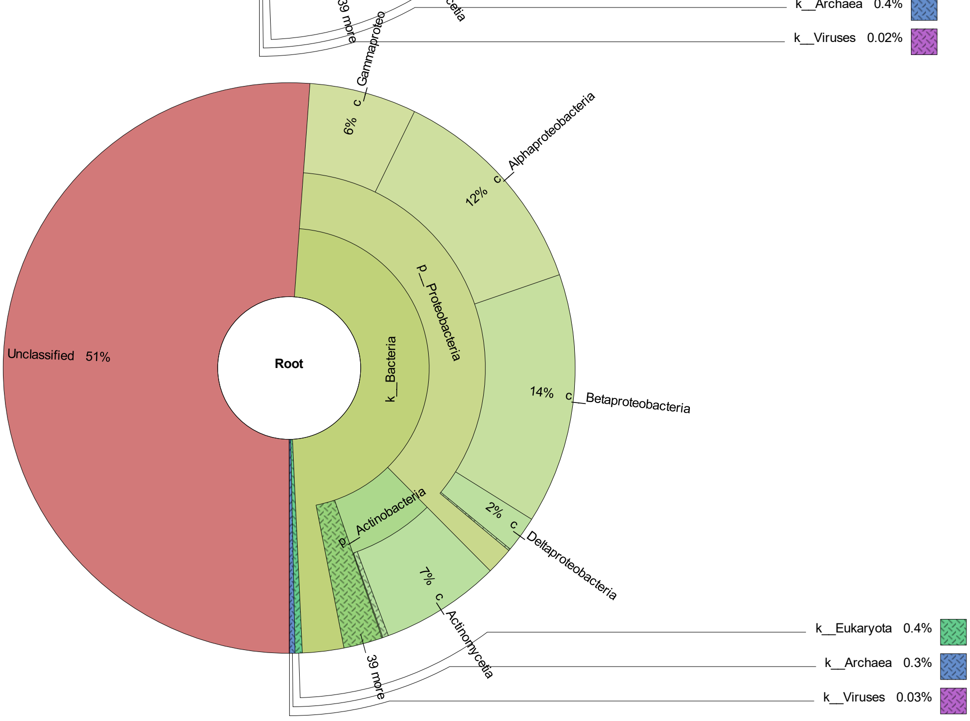

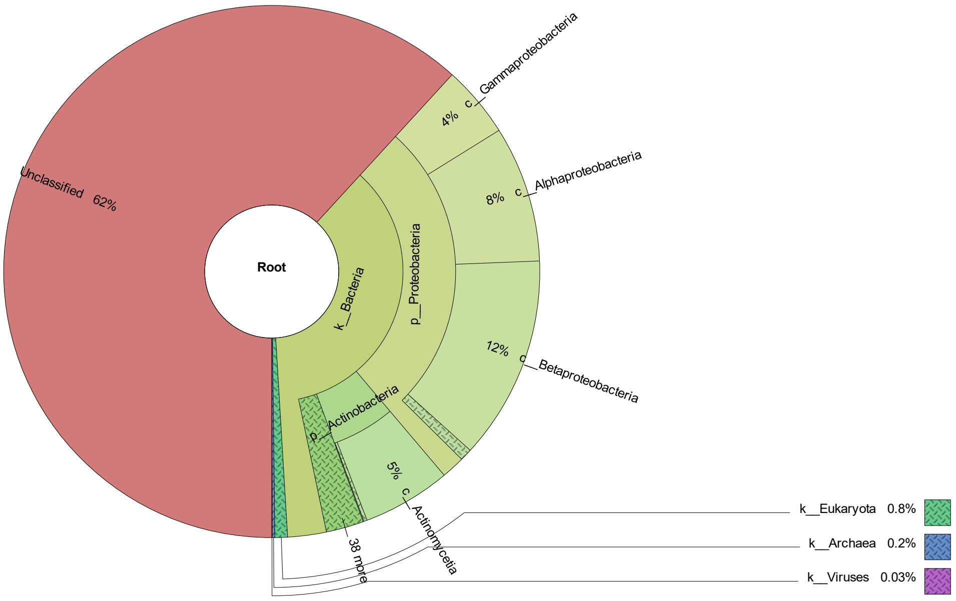

WWTP - Preliminary
28-25

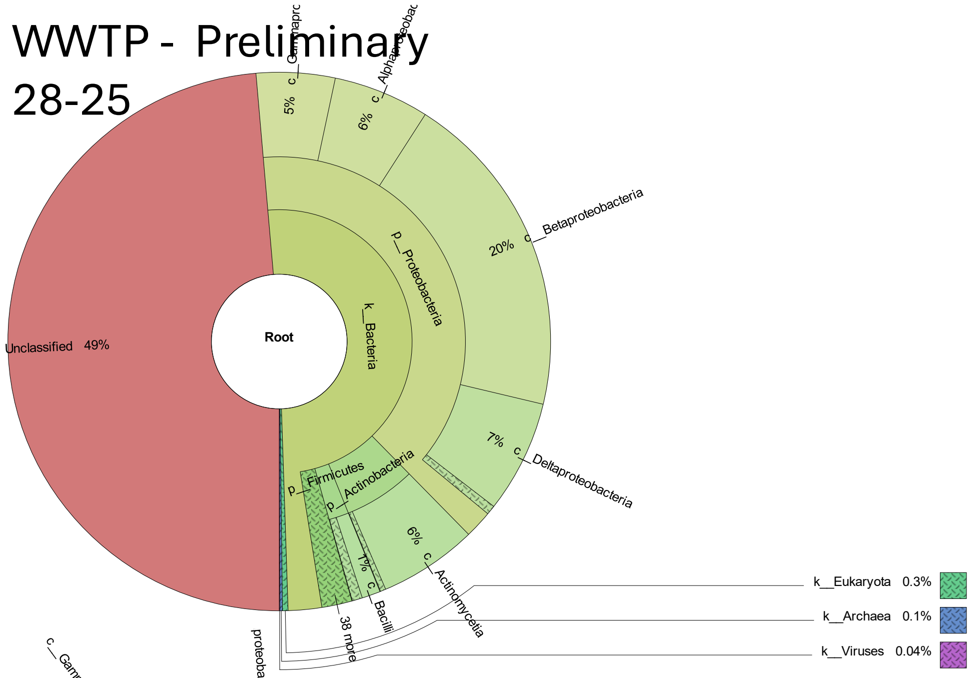

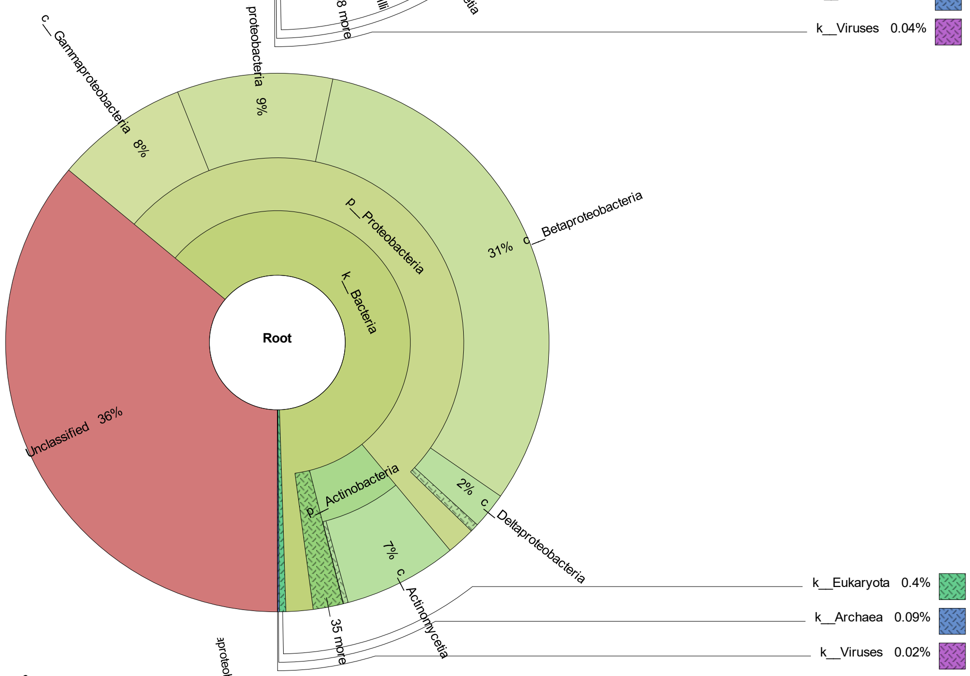

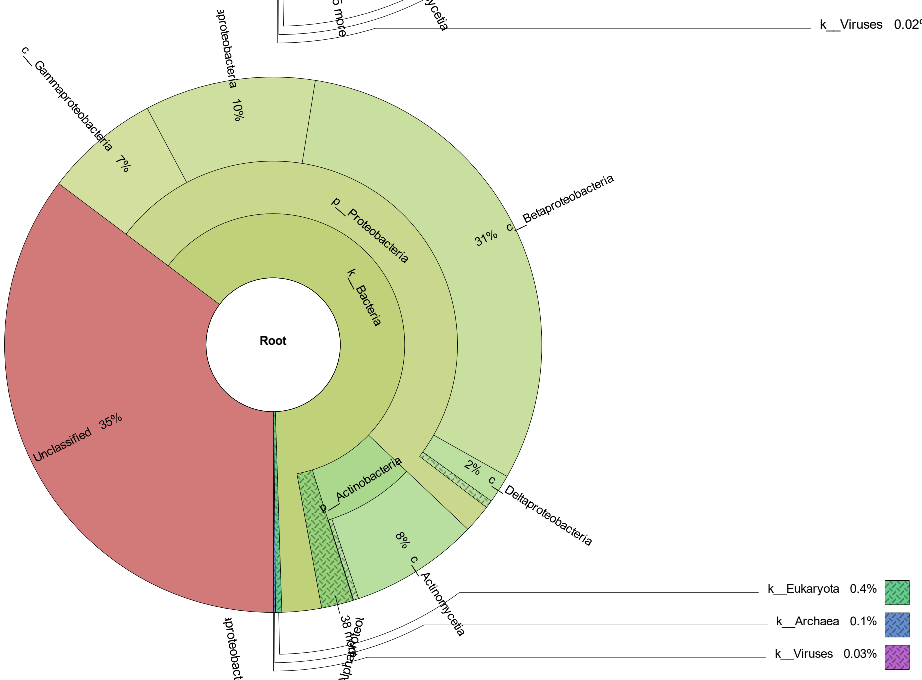

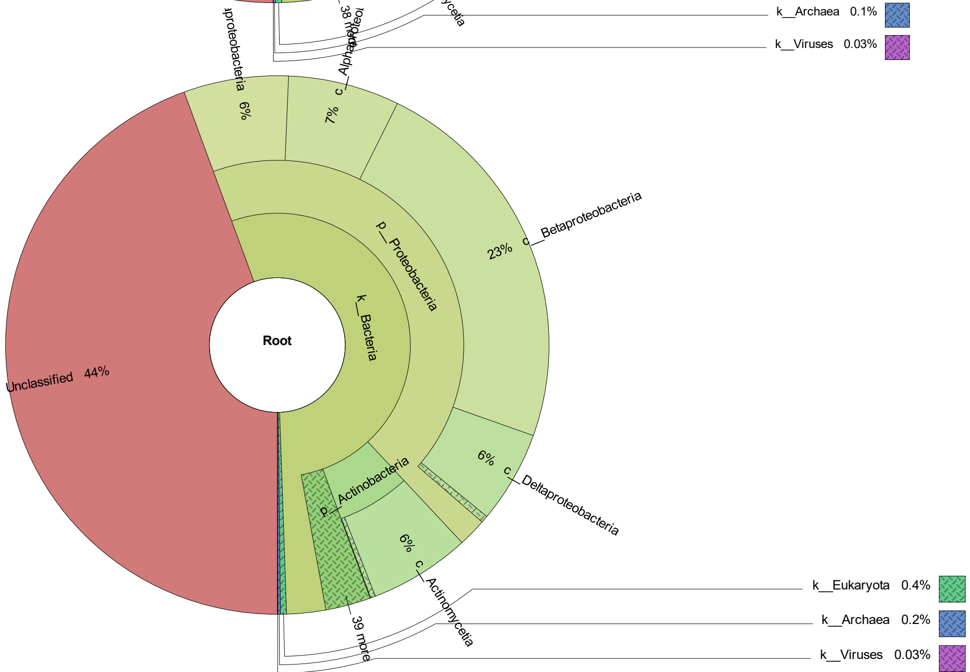

WWTP - Preliminary
24-21

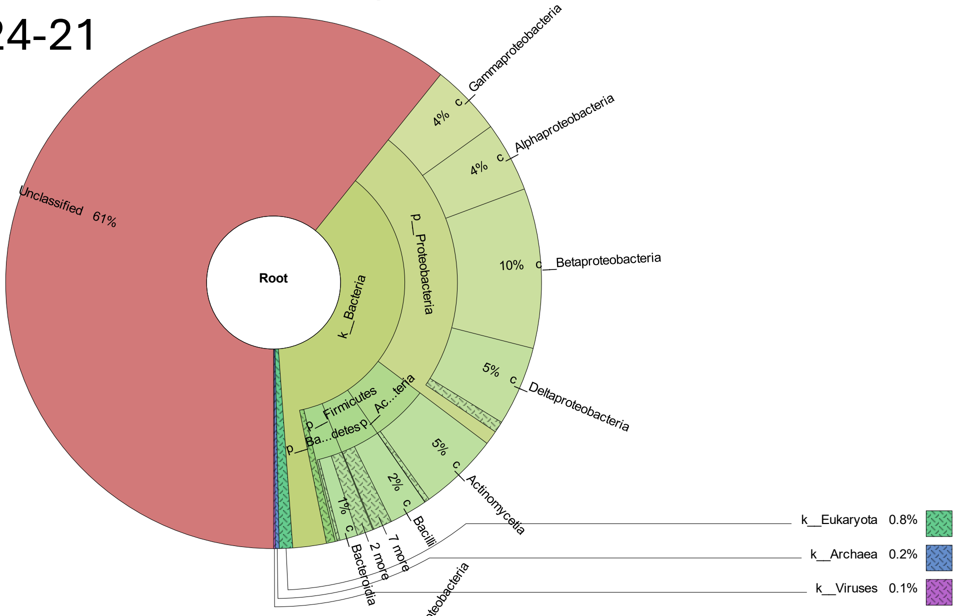

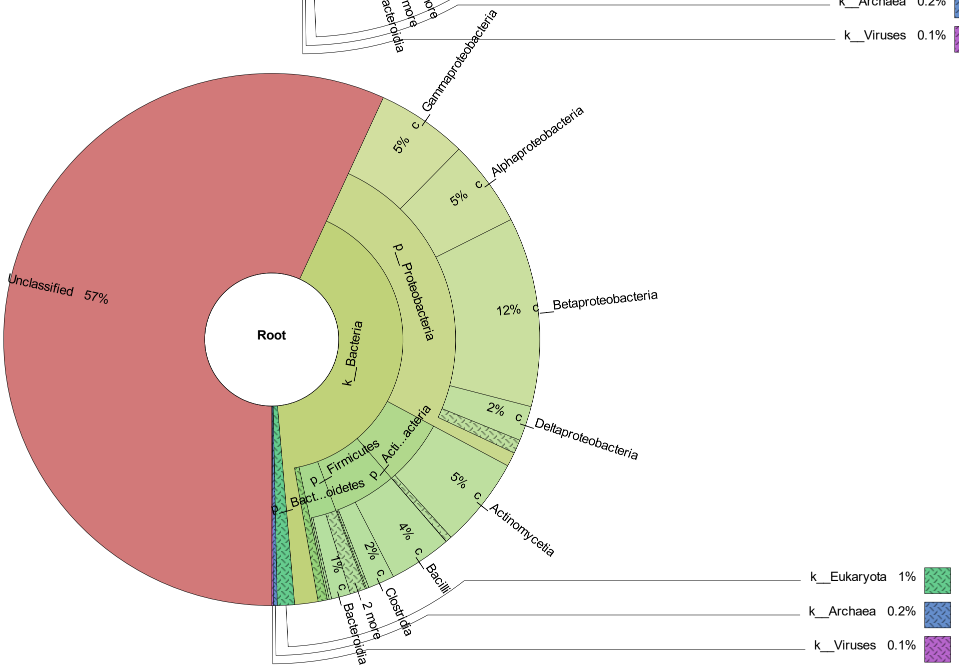

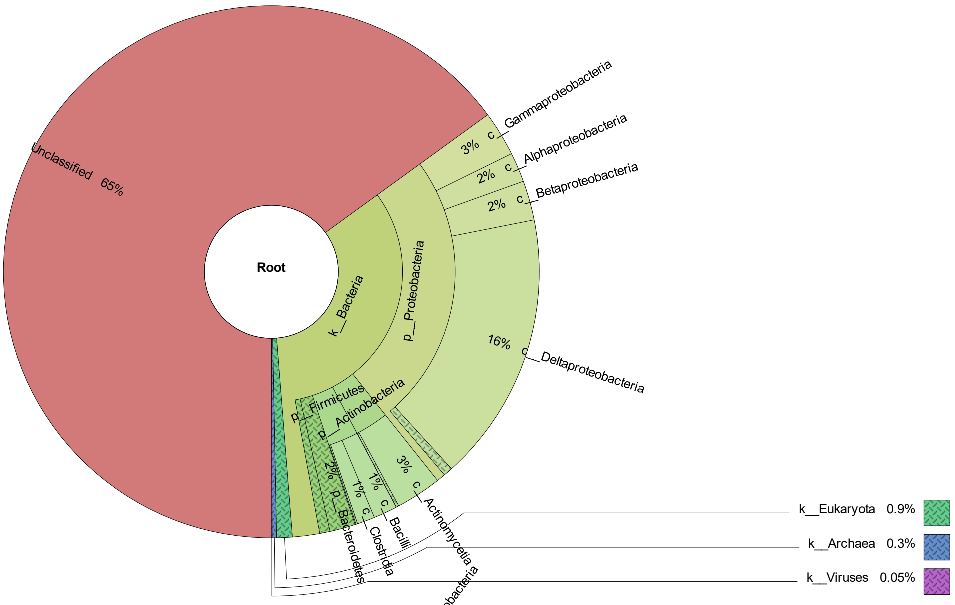

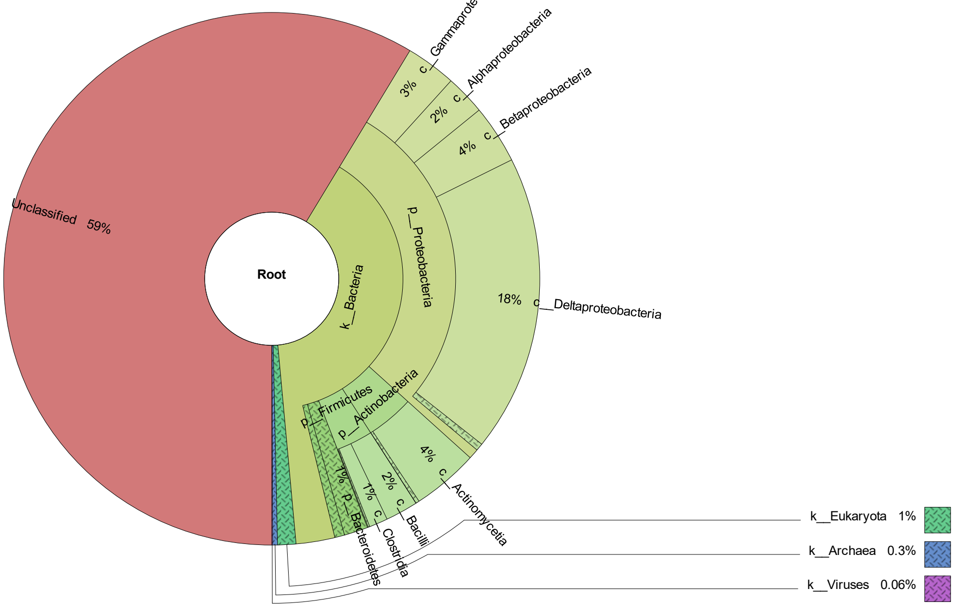

WWTP - Preliminary
20-19

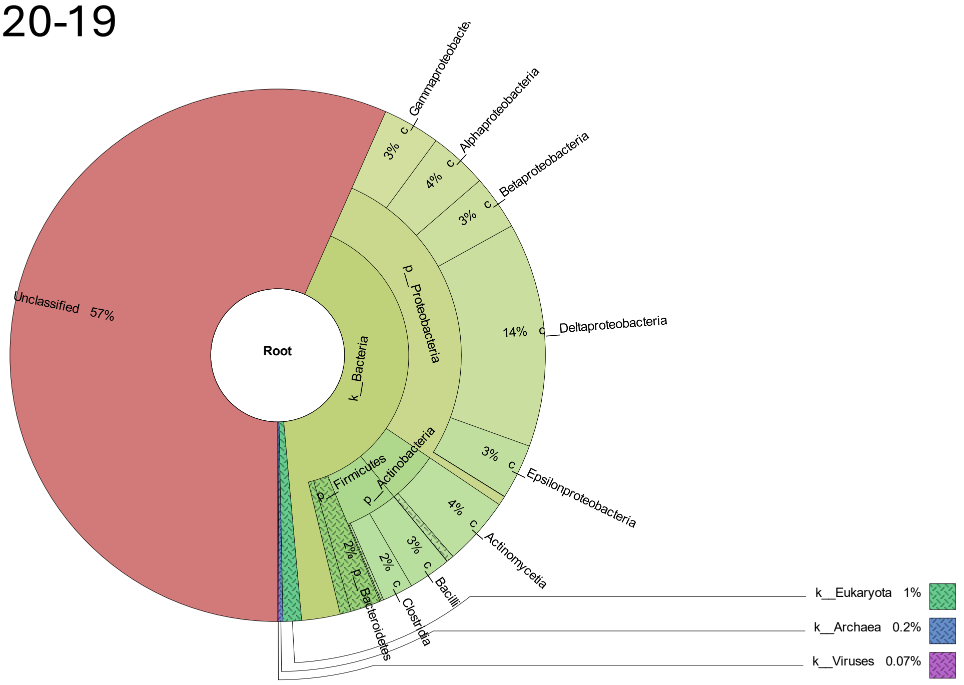

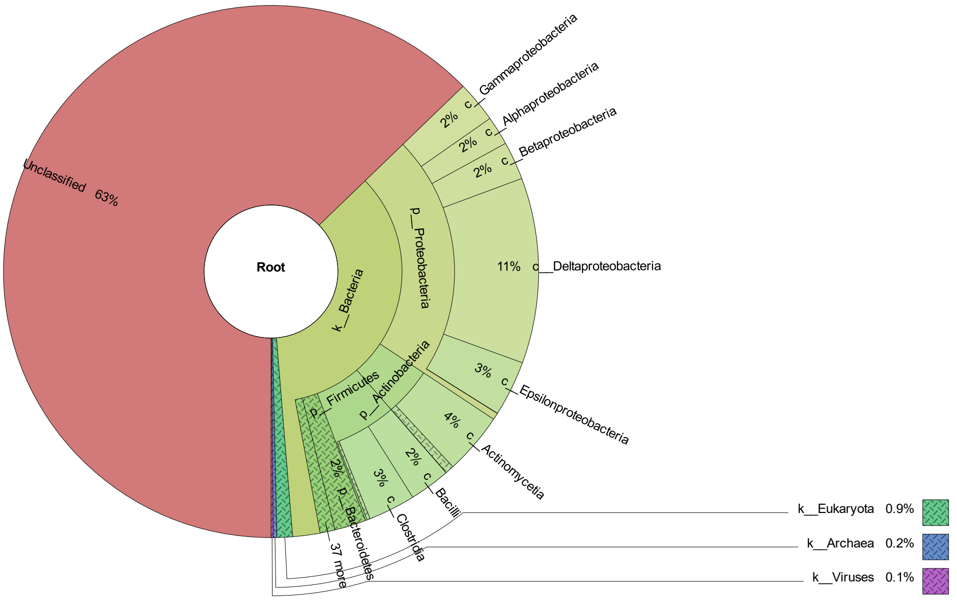

DWTP - Ozonation
18 – 16

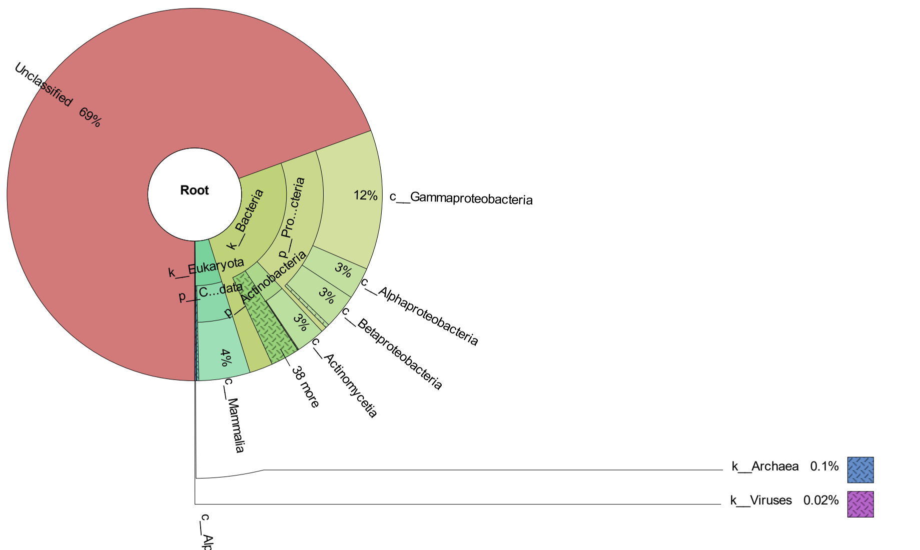

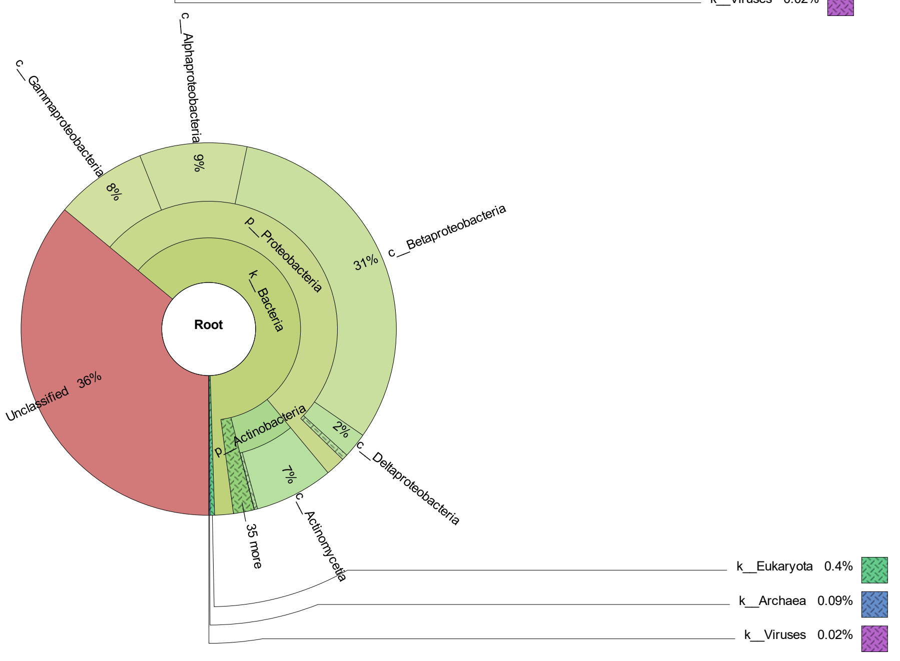

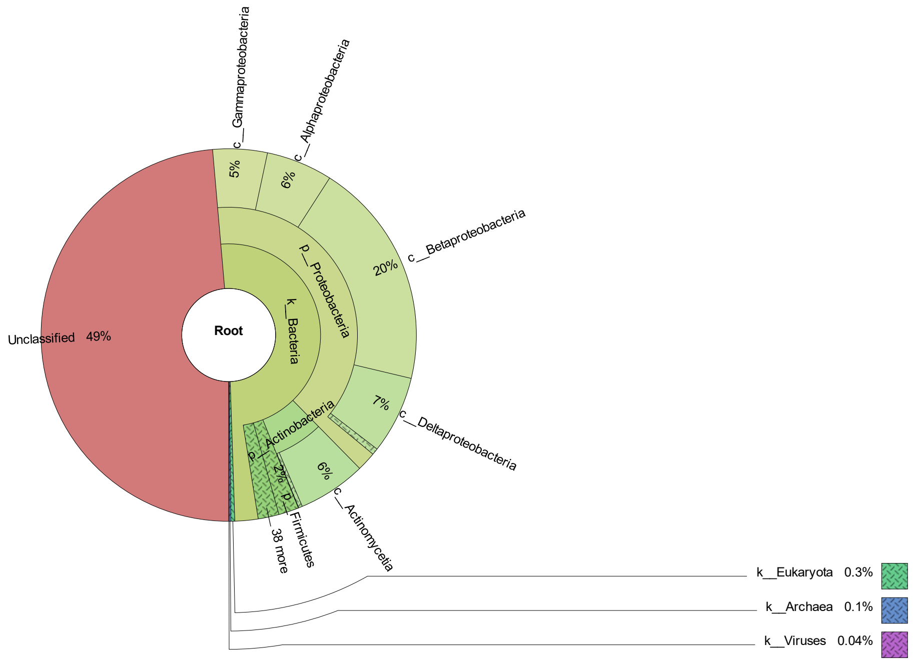

DWTP - Ozonation
15, 12

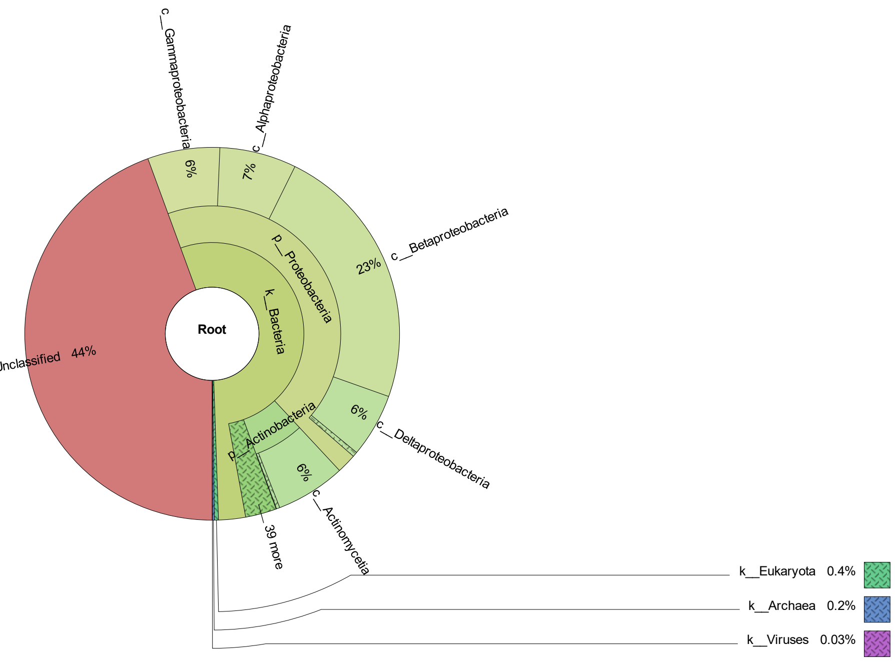

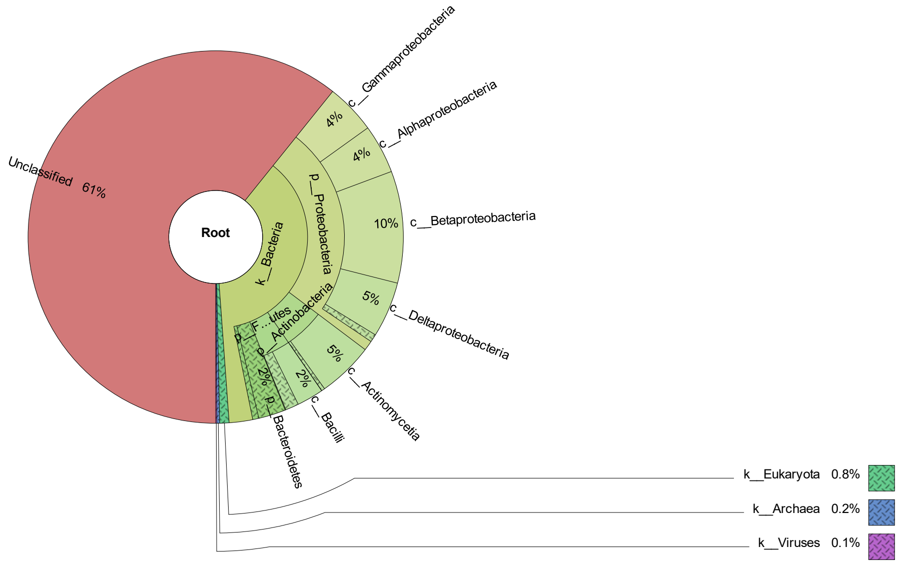

DWTP - Raw Water

14,13, 11 - 1

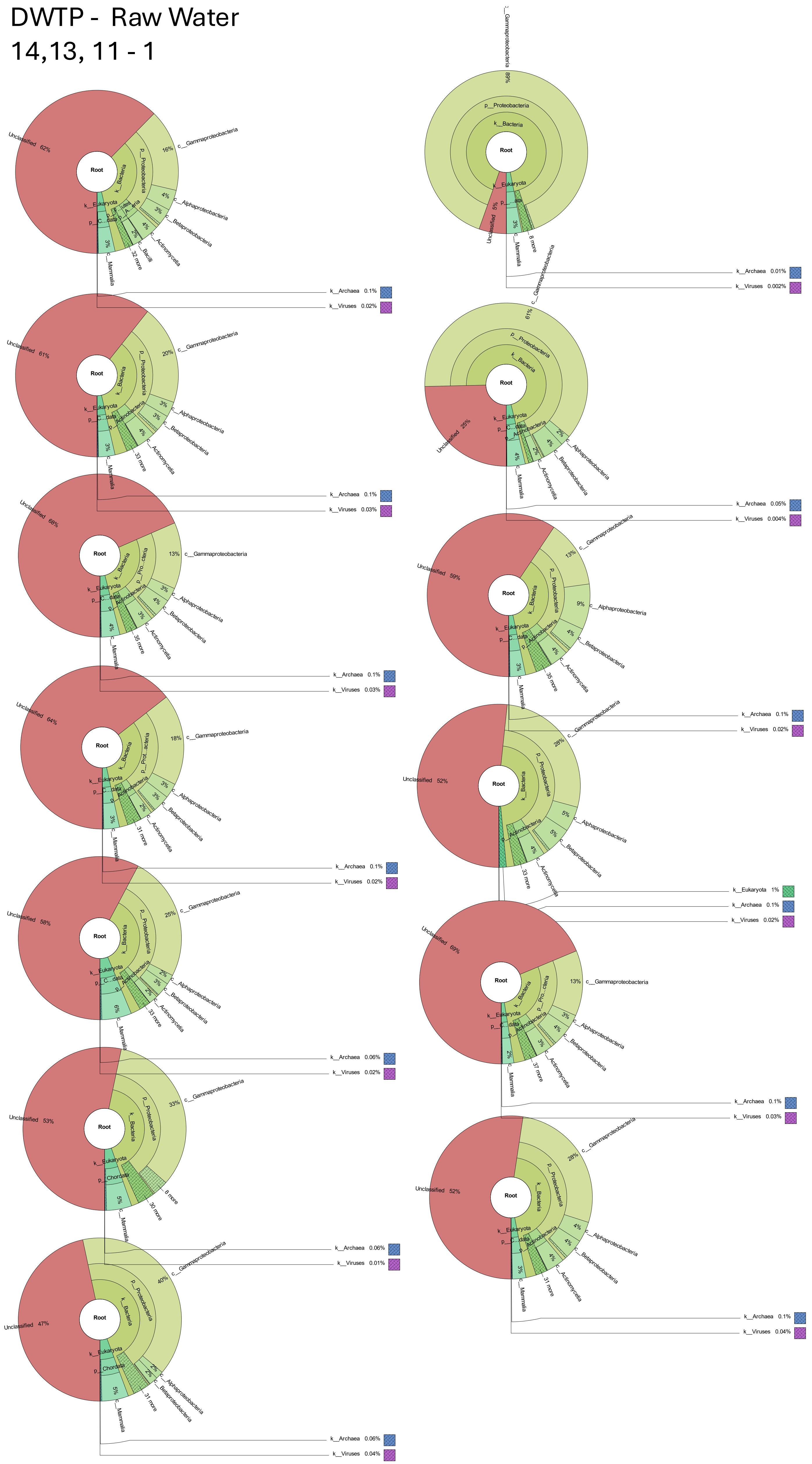

Supplement: Supplementary file 1 [file Data_Sheet_1.pdf]
